# Tailoring Successive Magnetic‐Dielectric Synergy for Enhancing Electromagnetic Absorption in Ultralong Heterostructure Chains Assisted by High Static Magnetic Field

**DOI:** 10.1002/advs.75440

**Published:** 2026-04-23

**Authors:** Zhuolin Li, Meilong Feng, Wenbo Gao, Tianxiang Zheng, Wenhao Lin, Yunbo Zhong

**Affiliations:** ^1^ State Key Laboratory of Materials for Advanced Nuclear Energy & School of Materials Science and Engineering Shanghai University Shanghai China

**Keywords:** electromagnetic absorption, high static magnetic field, interfacial polarization, magnetic‐dielectric synergy, one‐dimensional heterostructure

## Abstract

The dimensionality and size effects in magnetic‐dielectric composite governs a fundamental trade‐off between the intrinsic functionality and external tunability, which critically constrains the development of high‐performance electromagnetic (EM) materials with efficient magnetic‐dielectric synergy. Herein, we propose a magnetic‐field‐driven dual low‐dimension strategy to fabricate length programmable magnetic‐dielectric heterojunction chains, where one‐dimensional (1D) Fe_3_O_4_ chains were tightly encapsulated by the in situ grown two‐dimensional  MoS_2_ nanosheets. This strategy enables precise control over the chain length across micrometers to millimeters, as well as the surface defects, by deterministic regulation of the applied static magnetic field. Systematical theoretical simulations demonstrate that the high uniaxial anisotropy from 1D structure boosted magnetic response and the abundant Fe_3_O_4_/MoS_2_ heterointerfaces induced polarization enhancement jointly contribute to the unique successive synergistic loss mechanism in the 1D magnetic‐dielectric heterojunction chains. Eventually, the optimum 1D Fe_3_O_4_@MoS_2_ heterojunction chains with a record length of 2485.15 µm exhibit a broadband EM absorption performance with an effective absorption bandwidth of 5.5 GHz at a thin thickness of 1.8 mm, outperforming conventional counterparts. This study establishes a novel paradigm for crafting low‐dimensional magnetic‐dielectric heterostructure with tailored EM functionality, guiding the design of advanced EM materials for next‐generation flexible electronics.

## Graphical abstract

This work proposes a high static magnetic field (HSMF) driven dual‐low‐dimension strategy for fabricating magnetic‐dielectric heterostructure (Fe_3_O_4_@MoS_2_) chains with programmable lengths. The constructed successive magnetic‐dielectric synergy, arising from uniaxial anisotropy‐enhanced magnetic response and abundant heterointerfaces that boost polarization loss endows the resulting ultralong heterostructures with excellent multifunctionality: wideband effective electromagnetic absorption covering 5.5 GHz and sensitive thermal management capabilities.

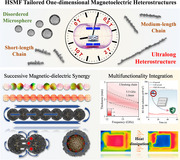

## 1 Introduction

The rapid advancement of cutting‐edge wireless communication technologies, including 5G and sub‐6G systems, has significantly accelerated progress in artificial intelligence, the Internet of Things, and next‐generation smart and flexible electronics [1, 2]. This technological evolution, however, brings a corresponding surge in electromagnetic (EM) radiation, which poses growing risks to both electrical devices and human health [3, 4]. Urgent demand exists for high‐performance EM absorption materials that can mitigate escalating interference by dissipating incident waves through dielectric and magnetic losses, as well as other loss mechanisms [5, 6, 7]. Benefiting from the potential synergy between dielectric and magnetic component (such as interface [8], coupling [9], and pinning effects [10]), magnetic‐dielectric composites outperform the traditional EM absorbers with a single loss pathway [11]. However, multicomponent integration, especially in three‐dimensional materials like bulks and particles, often suffers from intrinsic volume expansion and structural reconfiguration [11, 12]. These inevitable processes may cause material fracture and impedance mismatch, ultimately restricting the application of magnetic‐dielectric composites in miniature electronics [13, 14]. Consequently, rational low‐dimension design in components and structures are emerging as an effective strategy to address the mentioned considerations of magnetic‐dielectric composites but remains challenging.

Engineering low‐dimensional structure in magnetic materials is recognized as an essential strategy to withstand the restrictions of the Snoek limit [15, 16], which can effectively reconfigure the shape anisotropy thereby improving the involved demagnetization field [17]. The optimization of magnetic anisotropy eventually leads to a remarkable enhancement in intrinsic permeability and magnetic response abilities toward high‐frequency EM waves [18, 19]. Thus, extensive efforts have been devoted to low‐dimensional shape regulation, which mainly focus on elevating the in‐plane and uniaxial anisotropy of magnetic absorbers [20]. For example, Zhang et al. found that the flake geometry of two‐dimensional (2D) Fe_3_C microflakes could induce effective in‐plane anisotropy for improving magnetic loss [21]. Moreover, Gu et al. demonstrated that the one‐dimensional (1D) CoNi magnetic chains with uniaxial anisotropy exhibited stronger EM absorption capabilities compared to that with isotropic spherical structure [22]. Comparably, when facing the demands of flexibility and scalability during the application, 1D magnetic chains present more promising potential [23]. However, the dimensions for current 1D magnetic chains only ranges from tens of micrometers down to sub‐micrometer scales, are much smaller than the wavelengths of GHz EM waves with millimeters, leading to inadequate magnetic response thereby constraining the EM attenuation [18, 24, 25]. Therefore, precise regulation of magnetic chains from micrometers to millimeters scale to disclose the relationship between dimension and magnetic loss mechanism is urgently desired.

The spatial arrangements of dielectric and magnetic components in the magnetic‐dielectric heterogenous composite system also significantly affects the synergistic loss mechanism. In particular, employing dielectric materials to encapsulate the magnetic chains with high anisotropy can effectively relieve the undesired accumulation of surface electrons in the 1D structure, further optimizing the input impedance match [26, 27]. However, conventional nanoparticle‐based dielectric components are hard to achieve sufficient contact with the 1D magnetic chains owing to the space limitation [28], impeding the magnetic‐dielectric synergistic interaction. Consequently, introducing suitable dielectric components with low‐dimension structure to tightly coat the 1D magnetic chains is a feasible approach to address this issue. Recently, emerging low‐dimensional materials, including graphene [29], MXenes [30], and transition metal dichalcogenides (TMDs) [31], have garnered significant attention for tuning dielectric properties, due to their outstanding performance in electron transport and polarization response. Among them, TMDs‐based nanosheets possess the most impressive stability, compatibility and tunability, which has been widely applied as the dielectric components in the magnetoelectric composites EM materials [32, 33, 34]. Meanwhile, owing to their tunable electronic and band structure, the heterointerfaces built by magnetic components and TMDs can trigger adjustable interfacial polarization [35]. Therefore, encapsulating 2D TMDs on the surface of 1D magnetic chains to optimize EM dissipation performance of magnetic‐dielectric composites is effective but remains challenges due to the difficulty in precisely controlling their successive combination.

Based on these considerations, we proposed a dual low‐dimension strategy to fabricate 1D magnetic‐dielectric heterostructures with 2D MoS_2_ nanosheets decorated onto 1D Fe_3_O_4_ mcirochain (Fe_3_O_4_@MoS_2_) via a controllable magnetic‐field‐assisted assembly approach. Both of the length and the surface defects of the 1D Fe_3_O_4_@MoS_2_ heterostructures could be precisely programed by regulating the applied field strength, which presented an unique volcano‐shaped evolution tendency with the increasing field strength from 0 to 10 T and reached the peak at 2 T. Inspiringly, the longest 1D Fe_3_O_4_@MoS_2_ heterostructure chain with a recorded length of 2485.15 µm exhibited an impressive EM absorption performance: the effective absorption bandwidth covered 5.5 GHz at a thin thickness of 1.8 mm. Systematical mechanism analysis based on density functional theory, Multiphysics, and micromagnetic simulation disclosed that the ultralong 1D heterostructure remitted the Snoke limit for improving magnetic response and constructed successive magnetic‐dielectric synergistic interaction for enhancing EM loss. Eventually, such 1D magnetic‐dielectric chains filled liquid metal composites with a thickness of only 0.3 mm could achieve over 86.7% radiation reduction and efficient thermal conductivity, presenting promising application potential in the EM protection of the integrated electronic components.

## 2 Results and Discussion

### 2.1 Characterization and Microstructure Growth

**FIGURE 1:**
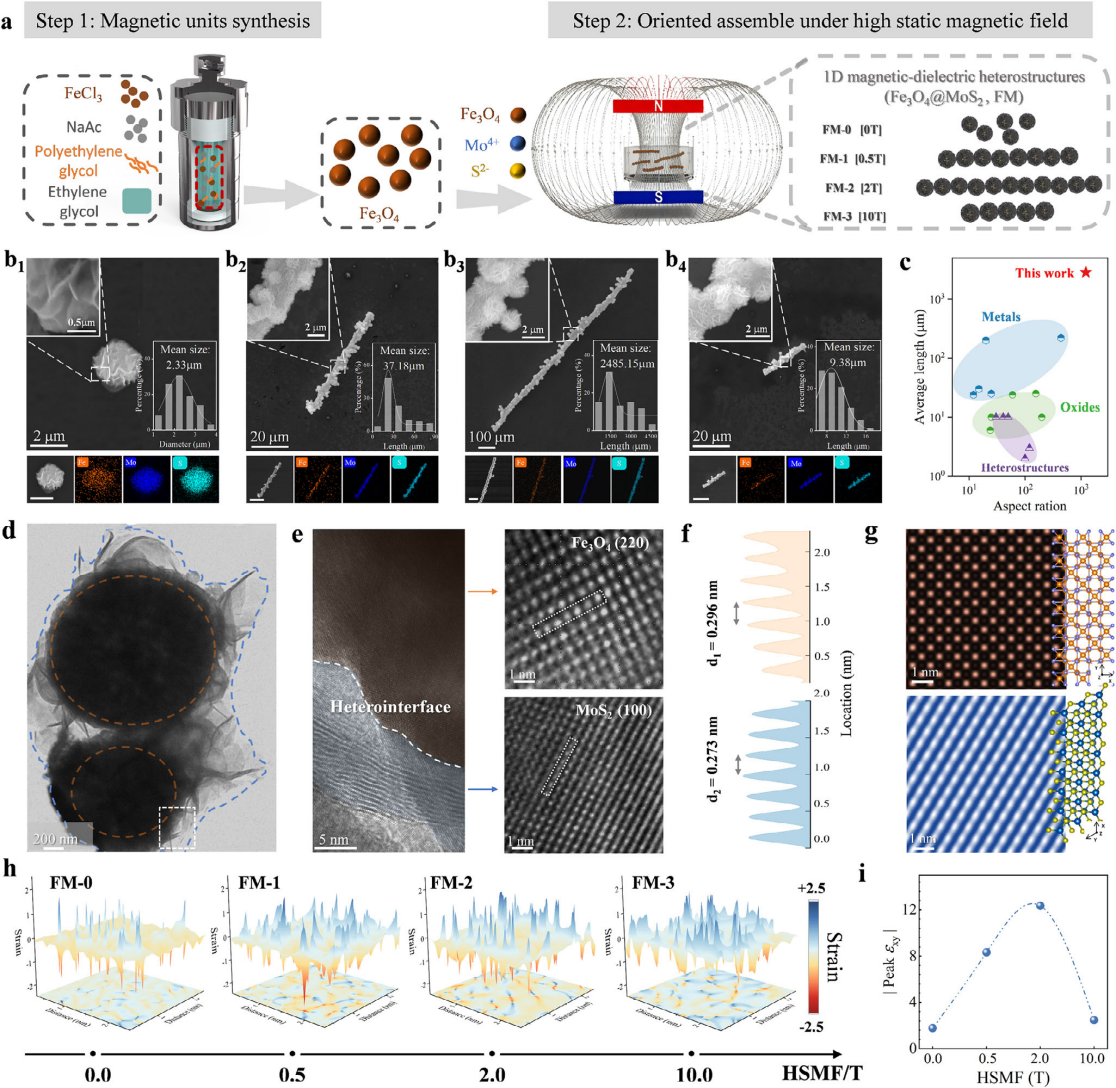
Fabrication and characterization of length‐tunable 1D magnetic‐dielectric heterostructure chains. (a) Schematic illustration for preparing the 1D Fe_3_O_4_@MoS_2_‐x (FM‐x) chains. SEM and EDS mapping of FM‐x specimens: (b_1_) FM‐0, (b_2_) FM‐1, (b_3_) FM‐2 and (b_4_) FM‐3, the insets were the statistics of the chain length and the magnified SEM images in the boxed regions, respectively. (c) Length comparison among reported heterostructure materials, the references were listed in Table S1. (d) TEM images of FM‐2 heterostructures. (e) HRTEM images of the selected area in d), and corresponding filtered results for identifying crystalline structure. (f) Measured lattice distance along the while boxes in filter HRTEM images. (g) QSTEM simulations of lattice structure, where the origin and bule parts were assigned to the Fe_3_O_4_ (220) and MoS_2_ (100), respectively. (h) 3D strain distribution map of MoS_2_ on the FM‐x surface, and (i) the effect of HSMF strength on the strain.

A schematic illustration of the synthesis of 1D magnetoelectric heterostructure chains with tunable lengths, denoted as FM‐x (Fe_3_O_4_@MoS_2_‐x, x = 0, 1, 2, 3), was shown in Figure 1a. First, well‐dispersed ferrimagnetic Fe_3_O_4_ microspheres with a uniform diameter of 2.33 µm were synthesized via a hydrothermal reaction. These magnetic units were then directed by an in situ high static magnetic field (HSMF) to assemble into 1D magnetic chains, and the lengths could be precisely programmed by tuning the applied magnetic field strength. Simultaneously, 2D MoS_2_ nanosheets were formed and tightly anchored onto the Fe_3_O_4_ surface through electrostatic interactions, stabilizing the 1D chain architecture and eventually constructing unique Fe_3_O_4_@MoS_2_ (FM) magnetoelectric heterojunctions. It should be noted that the FM heterostructures prepared under the magnetic field strength of 0, 0.5, 2 and 10 T were assigned to FM‐0, FM‐1, FM‐2, and FM‐3, respectively.

To identify the controllability of FM‐x heterostructures, the HSMF induced morphological variation were characterized by scanning electron microscopy (SEM). FM‐0 specimen prepared without HSMF remained the sphere‐like shape of the primary Fe_3_O_4_ microspheres, and its wrinkled surface corresponded to the coated MoS_2_ nanosheets, as is evident from the energy dispersive spectrometer (EDS) element mapping results (Figure 1b1; Figure S1). As the HSMF was applied, microchains with an average length of 37.18 µm and an aspect ratio of 16.22 were obtained in the FM‐1 (Figure 1b2), confirming that HSMF drove the formation of 1D structure. As the HMSF strength increased to 2 T, both of the length and aspect ratio in the FM‐2 further elevated to a recorded 2485.15 µm and 1066.59 (Figure 1b3), respectively. Notably, the diameters of the as‐prepared chains remained 2.33± 0.03 µm (Figure S2), which were similar to that of the FM‐0, indicating the oriented uniaxial alignment rather the random agglomeration by HSMF. Different from the conventional weak‐field behaviors, where the magnetic chain continuously lengthened along the increasing strength, this feature presented a sudden reduction as the strength beyond 2 T (Figures S3 and S4). This abnormal phenomenon in the HSMF might be ascribed to the supersaturation magnetization of the magnetic units by the external field [36, 37]. Eventually, the original chain‐like heterostructure remained but the length was deteriorated to 9.38 µm as the HSMF strength reached the maximum 10 T (Figure 1b4). Therefore, the lengths of these magnetic‐dielectric heterostructures presented a volcano‐shape evolution under HSMF, and the longest FM‐2 even outperformed majority of reported 1D materials (Figure 1c; Table S1).

The microstructure of Fe_3_O_4_@MoS_2_ heterostructure was revealed by the transmission electron microscopy (TEM). A typical core–shell structure was detected, where stacked MoS_2_ nanosheets tightly encapsulated the core Fe_3_O_4_ microsphere (Figure 1d). The unique heterointerfaces constructed by Fe_3_O_4_ and MoS_2_ were characterized using high‐resolution TEM (HRTEM, Figure 1e), and the lattice distances were measured to be 0.296 and 0.273 nm (Figure 1f), assigned to Fe_3_O_4_ (2 2 0) and MoS_2_ (1 0 0), respectively. Crystalline plane identification was also verified by the QSTEM simulations (Figure 1g), which are consistent with the HRTEM results, revealing the formation of the Fe_3_O_4_ (2 2 0)/MoS_2_ (1 0 0) interface. Additionally, the effect of HSMF on the outer MoS_2_ nanosheets was also investigated. As shown in the magnified surface regions of FM‐x specimens (the inset of Figure 1b), only slight variation in the morphologies and distribution of MoS_2_ nanosheet were detected. Then, their corresponding HRTEM images were recorded and analyzed with geometric phase analysis (GPA, Figure S5) to further reveal HSMF‐affected the microscopic lattice structures [38]. As shown in Figure 1h, obvious strain concentrations were detected in all MoS_2_ of FM‐x heterostructures, suggesting the existence of lattice defects. The derivate strain distribution histogram results (Figure S5a_3_–d_3_) illustrated the variation of peak strain values [39], which exhibited a similar trend to that of the chain length as the HSMF strength increased (Figure 1i). This correlation might be attributed to the intensified interfacial compression resulting from the assembly of magnetic units into elongated chains. These systematic microstructural characterization results demonstrated that both the length and surface crystalline defects in the 1D magnetoelectric heterostructure microchains could be precisely tailored by modulating the strength of HSMF.

**FIGURE 2:**
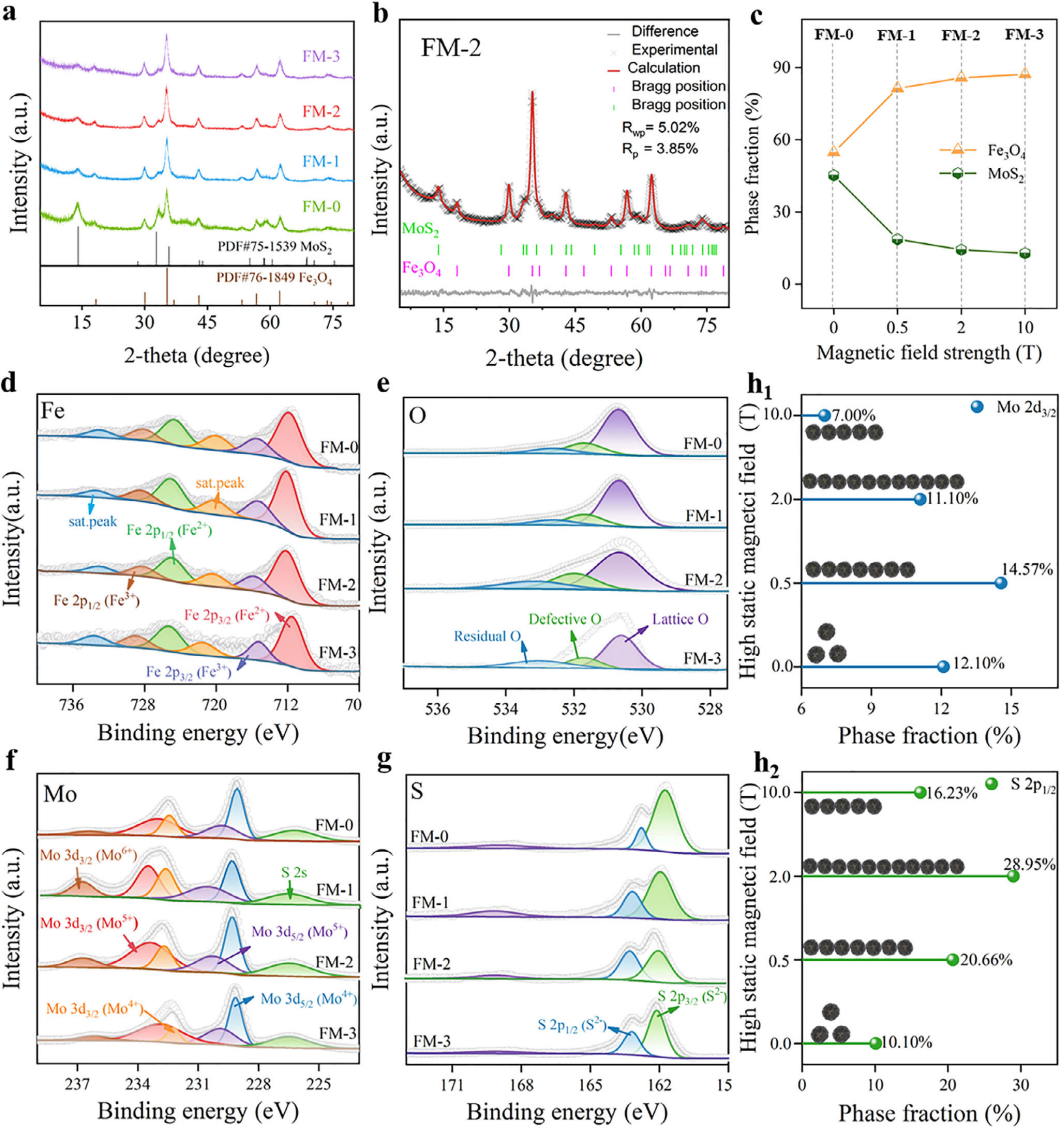
Effect of HSMF strength on phase composition and chemical state. (a) XRD patterns of FM‐x samples. (b) Refined XRD plots of FM‐2. (c) Phase fractions of Fe_3_O_4_ and MoS_2_ for FM‐x specimens. High‐resolution XPS spectra of (d) Fe 2p, (e) O 1s, (f) Mo 2p and (g) S 2p in FM‐x samples. Phase fraction percentage of (h_1_) Mo and (h_2_) S for MoS_2_ from deconvolution of XPS spectra in FM‐x specimens, respectively.

In addition, the effects of HSMF on the phase composition and elemental chemical environment of the FM‐x heterostructures were revealed by X‐ray diffraction (XRD) and X‐ray photoelectron spectroscopy (XPS). Obvious characteristic peaks at 18.28°, 30.06°, 35.41°, 43.04°, and 62.50° assigned to the (111), (220), (311), (400) and (440) planes of Fe_3_O_4_, respectively, and the peaks at 14.13° and 32.91° indexed to the (002) and (100) planes of MoS_2_, respectively, were identified in all FM‐x samples (Figure 2a), confirming the stable phase composition. The corresponding Rietveld XRD refinements were performed for a semi‐quantitative analysis of the variation in the phase composition ratio (Figure 2b; Table S2, and Figure S6). As evident from Figure 2c, the relative ratio of MoS_2_ and Fe_3_O_4_ decreased with increasing the applied HSMF, demonstrating that excessive magnetic interactions may inhibit the formation of MoS_2_ during the HSMF‐assisted hydrothermal process, likely by suppressing interatomic diffusion in a manner that scales with the strength of the applied HSMF [40]. The overall XPS spectra showed the existence of Fe, Mo, S and O (Figure S7), and the effect of the HSMF on their chemical states were analyzed using high‐resolution XPS. In the Fe 2p spectra (Figure 2d), characteristic peaks corresponding to Fe^2+^ and Fe^3+^ were observed for all FM‐x samples. The peaks at 711.5 and 725.3 eV were assigned to the 2p_3/2_ and 2p_1/2_ of Fe^2+^, respectively, while those at 715.2 and 728.8 eV corresponded to the 2p_3/2_ and 2p_1/2_ of Fe^3+^, respectively [41]. Neither the intensity nor the relative ratio of the characteristic Fe 2p peaks was significantly altered by increasing the HSMF strength. Similar variations were also observed in the O 1s spectra (Figure 2e), where the peaks located at 530.6, 531.7 and 533.2 eV were assigned to lattice oxygen, the surface oxygen vacancies and the unavoidable residual water molecules, respectively [42]. These results confirmed the chemical stability of magnetic Fe_3_O_4_ under the HSMF. In addition, the Mo 3d spectra (Figure 2f) exhibited characteristic peaks at 229.4 and 232.7 eV, correlated to the Mo^4+^ in 3d_5/2_ and 3d_3/2_, respectively, while the peaks located at 230.3 and 233.1 eV were matched the Mo^5+^ in 3d_5/2_ and 3d_3/2_, respectively. Furthermore, the peak at 236.8 eV corresponded to the Mo^6+^ in 3d_3/2_ due to the influence by HSMF during the formation of MoS_2_, and that at 226.5 eV was related to the S^2−^ state of MoS_2_ [43]. Notably, as illustrated in Figure 2h1, the phase fraction derived from the characteristic peaks of Mo 3d_3/2_ increased initially and then decreased with the increase in the HSMF strength. At the same time, the variations in S 2s were also observed in Figure 2g, where the peaks at 162.1 and 163.2 eV were assigned to S^2−^ 2p_3/2_ and S^2−^ 2p_1/2_, respectively [44]. Moreover, the phase fraction of the characteristic S 2p_1/2_ peaks decreased with decreasing chain length in Figure 2h_2_. The phase fraction variation of MoS_2_ with increasing HSMF aligned with the trend of the strain distribution, indicating that the HSMF intervention could effectively regulate MoS_2_ formation and its intrinsic defect properties. These physiochemical characterization results demonstrated the stable phase composition of the 1D FM magnetic‐dielectric heterostructures, and that the relative component ratio and involved vacancy states could also be regulated by the HSMF.

### 2.2 High Uniaxial Anisotropy Endowed Strong Magnetic Interaction

**FIGURE 3:**
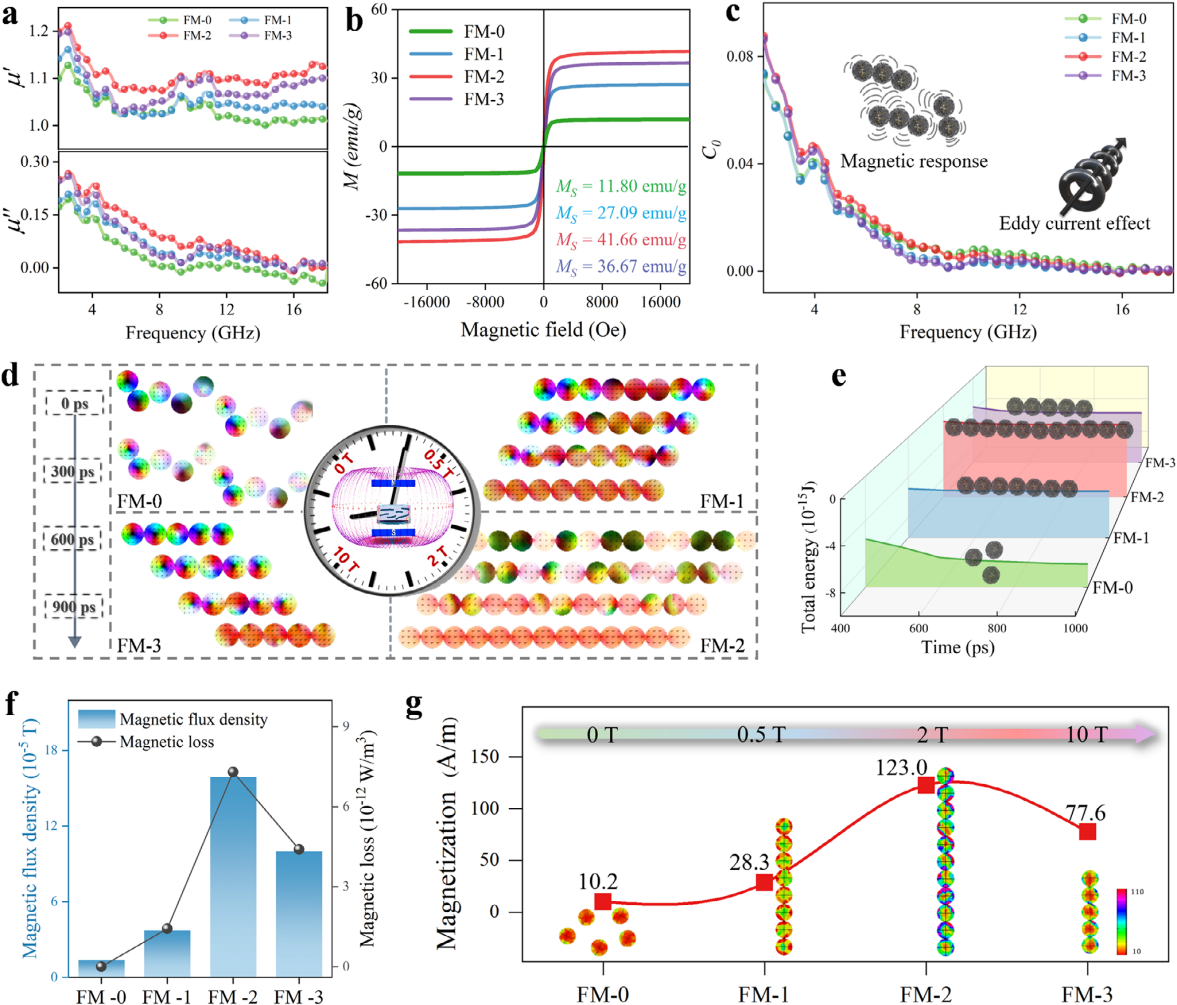
Length affected magnetic properties evolution. (a) Real (*µ'*) and imaginary (*µ''*) part of permeability. (b) Room temperature hysteresis loops in FM‐x samples. c) Profiles of *C*_0_ value along the frequency. (d) Micromagnetic simulated magnetic domain structures of FM‐x samples with different lengths and (e) corresponding total magnetic energy. Finite element simulated (f) variation of magnetic flux density and magnetic loss, and (g) corresponding magnetization values, the inset assigned to the distribution of magnetization.

The 1D chain‐like configuration exhibited great potential in increasing the anisotropy of magnetic materials for breaking Snoek limits, and further improving the intrinsic magnetic response behaviors toward the EM wave [15, 16]. To elucidate the magnetic properties of the 1D heterostructures, the complex permeability parameters of the FM‐x were analyzed. As shown in Figure 3a, a slight decline in the variation of the permeability with increasing frequency indicated that the FM‐x had a typical frequency dispersion property, implying the contribution of the magnetic dissipation loss mechanism. Generally, the real (μ′) and imaginary (μ′′) part of permeability represented the magnetic storage and loss ability, respectively [45, 46]. The μ′ values for the FM‐x samples at 2 GHz gradually increased with the chain lengths. This trend indicated that a longer chain structure enhanced the intrinsic magnetic characteristics owing to the excellent uniaxial anisotropy of the chain [22], with the longest‐chain FM‐2 exhibiting the most favorable magnetic energy storage capability among the series. Similar length‐affected variations were also observed in the μ′′, where the ultralong FM‐2 chain exhibited the highest μ′′ value at 2 GHz. This result indicated that the ultralong‐chain was conducive to improving the magnetic dissipation capability, which was mainly originated from high uniaxial anisotropy enhanced magnetic response. In addition, the complex permeability of the short‐chain (FM‐3) was higher than that of the medium‐chain (FM‐1). This result stems from the dual role of HSMF, which governs both chain growth and magnetization. Although a higher HSMF intensifies the magnetization of individual microspheres, it ultimately inhibits the assembly of the longest chains [47].

The saturation magnetization (*Ms*) as an important magnetic property was also investigated, which exhibited a volcano‐shaped variation along the increased HSMF (Figure 3b), increasing from 11.80 emu/g (FM‐0) to a maximum of 41.66 emu/g (FM‐2) and then decreasing to 36.67 emu/g (FM‐3), indicating the enhanced magnetic loss ability by increased magnetic interaction. The magnetic loss tangent (tan δ_μ_) of FM‐x series exhibited a declining trend with increasing frequency (Figure S8). At the initial measured frequency, the long‐chain FM‐2 showed the highest value (≈ 0.21), consistent with the superior magnetic loss capability. Additionally, the frequency dispersion was observed, with distinct resonance peaks near ∼9.7 and ∼10.9 GHz across the FM‐x samples, suggesting the contribution of natural resonance and exchange resonance toward the magnetic loss [48]. The magnetic loss mechanism was further evaluated by the eddy‐current coefficient, *C*_0_ (Figure 3c, calculated by Equation S4). The *C*_0_ curves of the FM‐x systems flattened with increasing frequency and eventually approached constant values in the high‐frequency range. This behavior indicates that the eddy‐current loss mechanism was also involved, thereby enhancing the overall magnetic response and magnetic loss capability [49].

To gain deeper insight into the magnetic response behaviors in the length‐controlled 1D heterostructure, micromagnetic simulations were performed to reveal the evolution of the total energy and migration of magnetic moments [18, 50]. The cyclical variations of total energy and magnetic energy dissipation in four simplified models were investigated. These models simulated the evolution of magnetic domain flipping in FM‐x heterostructures (Figure 3d), where random magnetic moment flipping within individual microparticles were detected in FM‐0 microsphere. In contrast, when these magnetic spheres were assembled into successive chains with uniaxial anisotropy, their magnetic domains align uniformly along the chain direction, indicating that the domains experienced strong magnetic coupling in response to the altered external field. Additionally, the total energy of the anisotropic chain exceeded that of the dispersed microspheres (Figure 3e), with the FM‐2 ultralong chain achieving the maximum value. This result confirmed that the chain structure generated a stronger magnetic response, leading to exceptional magnetic attenuation capability. Collectively, these simulations revealed that the dynamic behavior of the magnetic domains was a key factor governing both the cyclical energy variation and the overall magnetic loss in these chain‐structured materials.

Then, finite element simulation was employed to investigate the interaction between the FM‐x specimens and incident EM wave and to examine the evolution of magnetic properties [48, 49]. The simulated field distributions elucidated the underlying coupling mechanism (Figure S9), and the variation in surface color represented the corresponding changes in magnetic flux density and magnetic loss toward EM field. These results demonstrated that the FM‐x samples generated significant magnetic interactions with the incident EM waves. Additionally, the magnetic flux density and magnetic loss initially reached a minimum for the FM‐0 microspheres (Figure 3f), then attained a maximum for the ultralong‐chain FM‐2, and subsequently decreased for the short‐chain FM‐3. A consistent trend was observed in the magnetization (Figure 3g), the pronounced intensity assigned to an increased magnetization, further verifying that the chain length was conducive to magnetic loss capability. Therefore, the design of the ultralong magnetic‐dielectric heterojunction chain endowed the FM‐2 with high uniaxial anisotropy, which contributed a superior magnetic loss.

### 2.3 Enhanced Dielectric Loss by Successive Heterointerface Polarization

**FIGURE 4:**
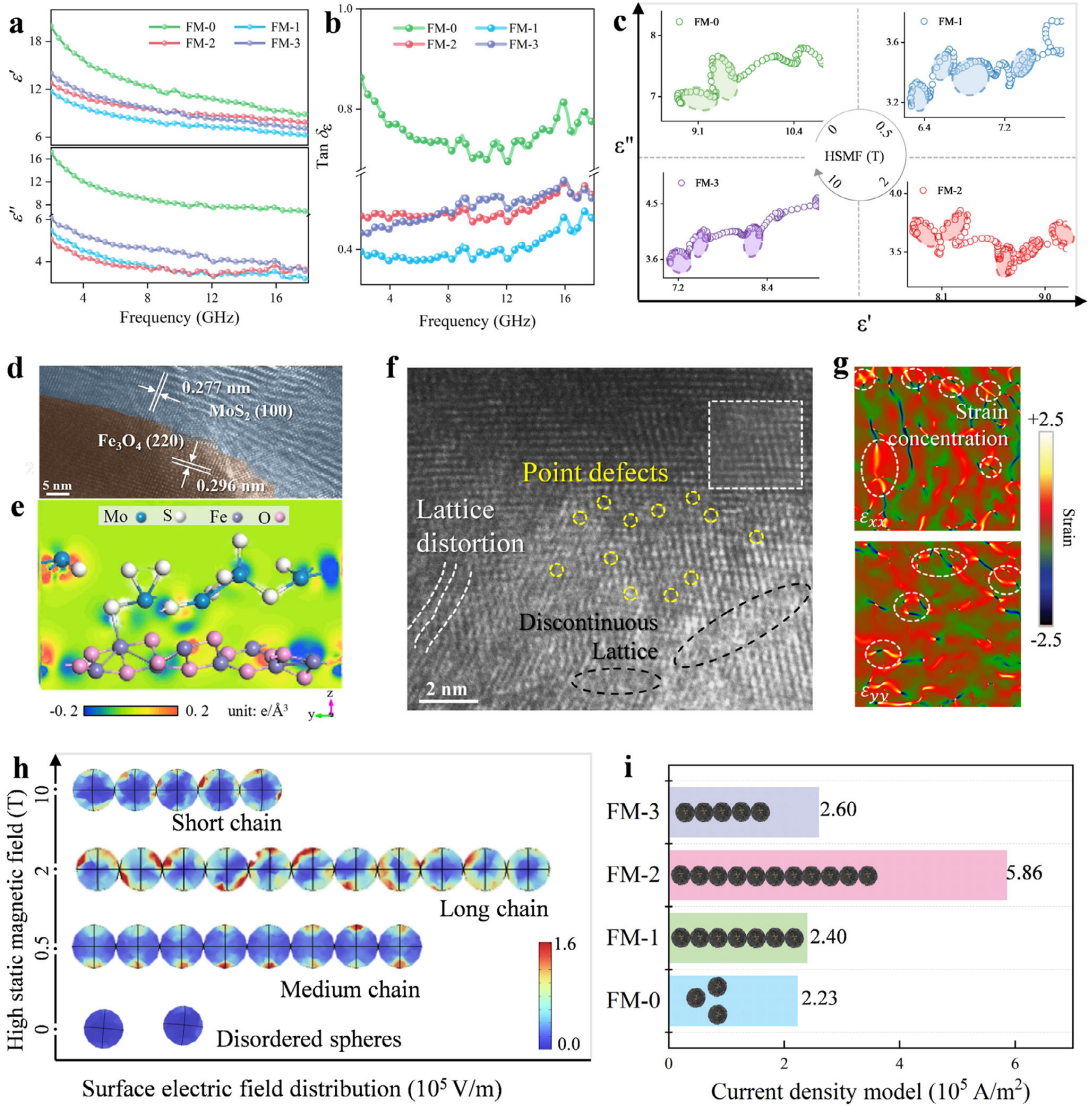
Analysis of successive magnetic‐dielectric synergistic loss mechanism. (a) Real (ε') and imaginary (ε'') permittivity of FM‐x samples. (b) Tangent values of dielectric loss. (c) Cole–Cole curves of FM‐x samples. (d) HRTEM image of FM‐2 magnetic‐dielectric heterointerface, the orange and bule regions were assigned to Fe_3_O_4_ and MoS_2_, respectively. (e) Calculated charge density difference of the heterointerface built by Fe_3_O_4_ (220) and MoS_2_ (100). (f) HRTEM image of the Fe_3_O_4_ in FM‐2. (g) GPA results of the white dotted box region in (f). Finite elements method simulated (h) surface electric field distribution of FM‐x samples with different length, and (i) corresponding current density.

Decorating abundant MoS_2_ nanosheets on 1D magnetic chain surfaces not only stabilized the 1D structure but also optimized the electronic properties. The introduction of the HSMF modulated both the length and surface defects, potentially affecting the eventual dielectric ability toward EM wave. Therefore, to clarify the correlation between the continuous heterointerface in magnetic‐dielectric chain and the dielectric loss mechanism, the complex permittivity parameters were analyzed. As shown in Figure 4a, both of the real (ε′) and imaginary (ε′′) parts of permittivity in FM‐x samples presented typical frequency dispersion property, reflected by the continuous decreased values aligned with the increased frequency [51], indicating the involvement of dielectric loss mechanism. Additionally, pronounced resonance peaks were observed in the ε′′ and tan δ_ε_ plots of the FM‐x heterostructures (Figure 4b), suggesting the existence of polarization relaxation. Due to the abundant successive heterointerfaces and surface defects in the 1D chain FM‐x specimens, interfacial and dipoles polarization could be induced for dielectric loss [45, 50]. Then, Cole‐Cole curves were applied to further investigate the length‐affected polarization loss capability, where a Cole–Cole semicircle represented the existence of potential polarization relaxation [51, 52]. As shown in Figure 4c, the numbers of Cole‐Cole semicircles were increased as the chain lengthened, and the longest FM‐2 heterojunction chain exhibited the most pronounced semicircular profile (Figure S10), signifying the polarization dominated dielectric loss in dissipating the incident EM waves.

To uncover the enhanced polarization loss in ultralong FM‐2 heterostructure chain, the involved magnetic‐dielectric microstructures were systematically analyzed by experimental visualization and theoretical calculation. Based on the observed heterointerface built by Fe_3_O_4_ (2 2 0) and MoS_2_ (1 0 0) planes (Figure 4d), the interfacial charge density difference distributions were calculated by density functional theory (DFT) [53]. As shown in Figure 4e, obvious potential differences existed at the Fe_3_O_4_/MoS_2_ interface, which could employ as a capacitor–like electronic structure for dissipating the altered EM field [54]. Additionally, the HSMF‐driven combination of Fe_3_O_4_ and MoS_2_ also caused atomic reconstruction and numerous defects, including point defects, lattice distortions, and discontinuities in core Fe_3_O_4_ (Figure 4f). These high density of lattice defects triggered significant localized strain concentration (Figure 4g), and induced dipoles to realign under an applied field, thereby providing an additional loss pathway for dissipating EM energy [55]. Therefore, such successive heterostructure facilitated a synergistic enhancement of the dielectric loss for EM dissipation.

Finite element simulations of EM wave interactions with the FM‐x chain structures were performed to further clarify the significance of successive heterointerfaces in dielectric loss [56]. The evolution of the surface electric field distribution, which directly reflected the response of the interfacial polarization, followed a positive advancement with the increased chain length (Figure 4h). The long chain structure amplified the surface electric field distribution, leading to pronounced electron accumulation, which suggested stronger interfacial polarization loss than that in the non‐chain structure systems. Additionally, similar variation tendency was detected in current density (Figure 4i), where FM‐2 exhibited a higher current density, validating that chain length could effectively accelerate electron transport from the incident EM wave. These simulation results emphasized that the 1D ultralong chain architecture significantly enhances the localized electric field response and polarization relaxation, which effectively promoted the dielectric dissipation toward incident EM waves, further contributing to the final EM absorption performance.

### 2.4 EM Wave Absorption Application

**FIGURE 5:**
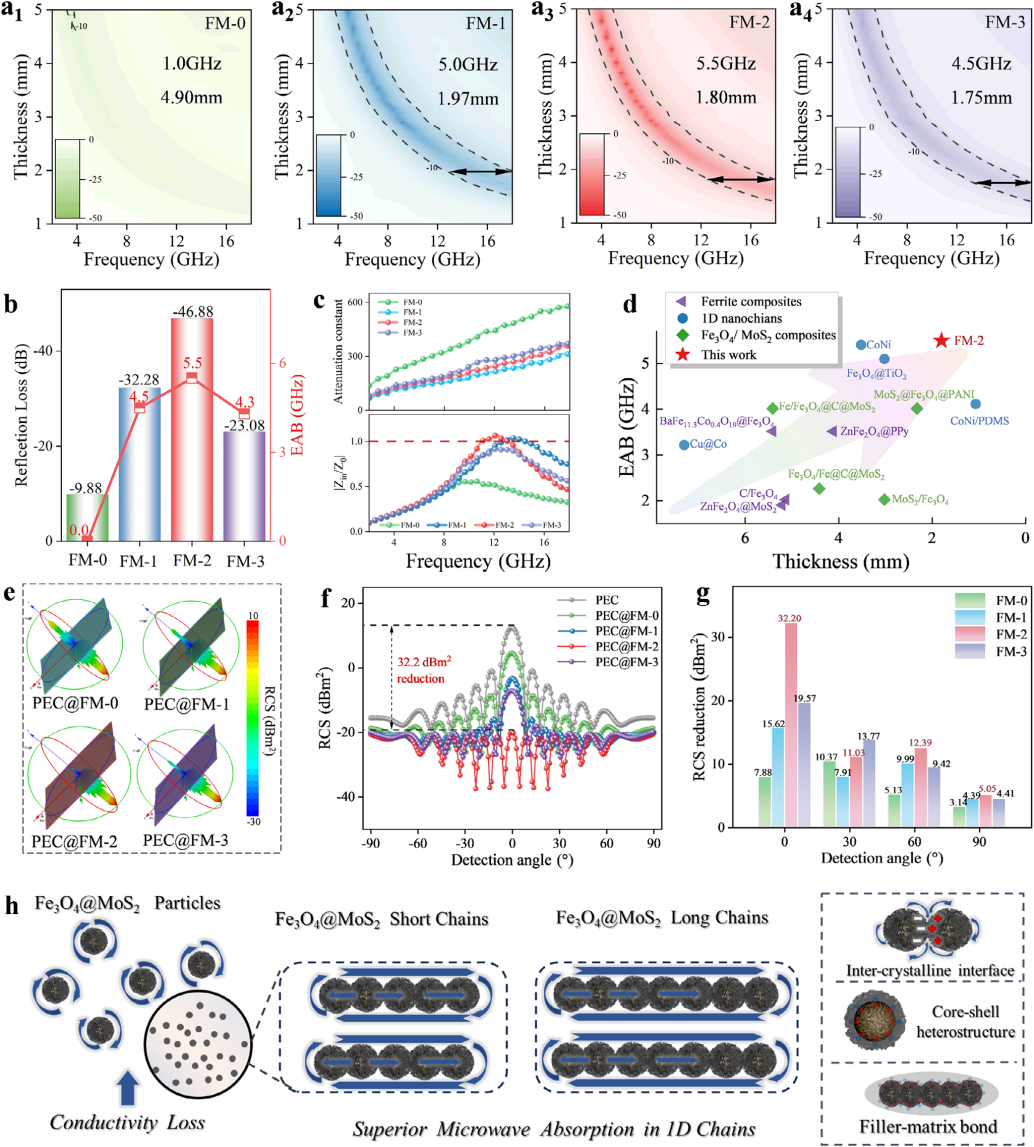
EM wave absorption performance. 2D RL maps for (a_1_) FM‐0, (a_2_) FM‐1, (a_3_) FM‐2, and (a_4_) FM‐3. (b) Comparison of the RL and EAB values at a thickness of 1.8 mm. (c) Attenuation constant values and the |*Z_in_*/*Z*_0_| plots at their optimum thickness. (d) EAB comparison among reported analogous absorbers, the related references were listed in Table S3. (e) 3D RCS patterns of PEC plate coated with FM‐x samples, and (f) corresponding 1D RCS profiles. (g) Effect of detection angles on the RCS reduction. (h) Schematical summarization of loss mechanism contributed to the superior EM absorption performance in the ultralong magnetic‐dielectric heterostructure chains.

Benefiting from the high uniaxial anisotropy reinforced magnetic response and the abundant successive‐heterointerfaces induced polarization enhancement, the ultralong magnetic‐dielectric heterostructure chain possessed a promising magnetic‐dielectric synergistic loss ability in EM absorption. Thus, the performance indexes including reflection loss (*RL*), effective absorption bandwidth (EAB, defined as the frequency range where *RL*  ≤   − 10 dB), and thickness, were systematically evaluated across the 2–18 GHz band [55]. In the 2D color *RL* maps (Figure 5a), the magnetic–dielectric heterojunction chains exhibited relatively a wider EAB under a thinner thickness than that of the FM‐0 microspheres. Specifically, the FM‐0 exhibited the weakest performance, with an EAB of only 1.0 GHz at 4.90 mm. In contrast, the chain‐like specimens showed significant absorption performance enhancement with the increased length, where their optimum EAB values evolved from 4.5 GHz in short‐chain FM‐3, to 5.0 GHz in medium‐chain FM‐1, and the widest 5.5 GHz in long‐chain FM‐2. In addition, the successive magnetic‐dielectric synergistic loss mechanism also endowed the chain heterostructure with impressive *RL* values (Figure 5b). The ultralong‐chain FM‐2 not only achieved a superior RL of −46.88 dB at a thin thickness of 1.8 mm but also demonstrated consistently better performance across the entire tested thickness range than others (Figure S11).

Then, the attenuation constant and impedance matching were evaluated to determine the effectiveness of EM absorption. Figure 5c showed that the attenuation constant decreased when a chain structure was formed. While a high attenuation constant typically indicated strong dissipation capacity, it alone cannot ensure high absorption performance because the effective loss required incident waves to first penetrate the material. Therefore, optimal impedance matching was an essential prerequisite for high‐performance EM absorbers. The impedance matching was reflected by the value of |*Z_in_*/*Z*_0_|, with a value closest to 1 representing ideal conditions [57]. As shown in Figure 5c and Figure S12, the |*Z_in_*/*Z*_0_| profiles demonstrated the most satisfying impedance matching of ultralong‐chain FM‐2 sample at its optimum thickness. This favorable impedance allows for deeper wave penetration and more efficient subsequent attenuation. Consequently, the combination of optimal impedance matching and superior intrinsic dissipation capability in the FM‐2 ultralong chains collectively enabled the impressive absorption performance with a broad EAB, and strong *RL* at a thin thickness, outperforming majority of reported analogous EM absorbers (Figure 5d).

To evaluate their practical applicability, the EM performance of FM‐x heterostructure coatings on perfect electric conductor (PEC) substrates was assessed via radar cross section (RCS) simulations under far‐field plane wave excitation [58]. 3D RCS results showed that all FM‐x coated plates exhibited suppressed backscattering compared to the bare PEC plate (Figure 5e and Figure S13). And these plates coated with chain structured FM‐x displayed significantly lower scattering than that of the FM‐0 coated plate. This result demonstrated the superior radar stealth property of the long chain architecture over the 0D microspheres, which was consistent with the RL properties. Additionally, Figure 5f showed the related 1D RCS values under the angular distribution from ‐90° to 90°. Notably, FM‐2 coatings exhibited the most significant RCS reduction, achieving a maximal reduction of 32.2 dBm^2^ at an incident angle of 0°, confirming its exceptional EM wave attenuation [59]. And the calculated RCS reduction values under four characteristic angles showed that the FM‐2 could maintain effective scattering protection toward the EM wave with different incident angles (Figure 5g). As summarized in Figure 5h, the outstanding performance in ultralong FM‐2 chain was mainly originated from the enhanced magnetic–dielectric synergy: on the one hand, multiple distinct interfaces (inter–crystalline, core–shell, and filler–matrix boundaries) strengthened the interfacial polarization loss [60]; on the other hand, the ultralong uniaxial anisotropy optimizes natural resonance and exchange coupling, thereby boosting the magnetic response. These results further confirmed the feasibility and significance of engineering successive low‐dimensional heterostructures to improve EM wave absorption performance.

### 2.5 Practical Application of FM‐x‐Based EM Multifunctional Films

**FIGURE 6:**
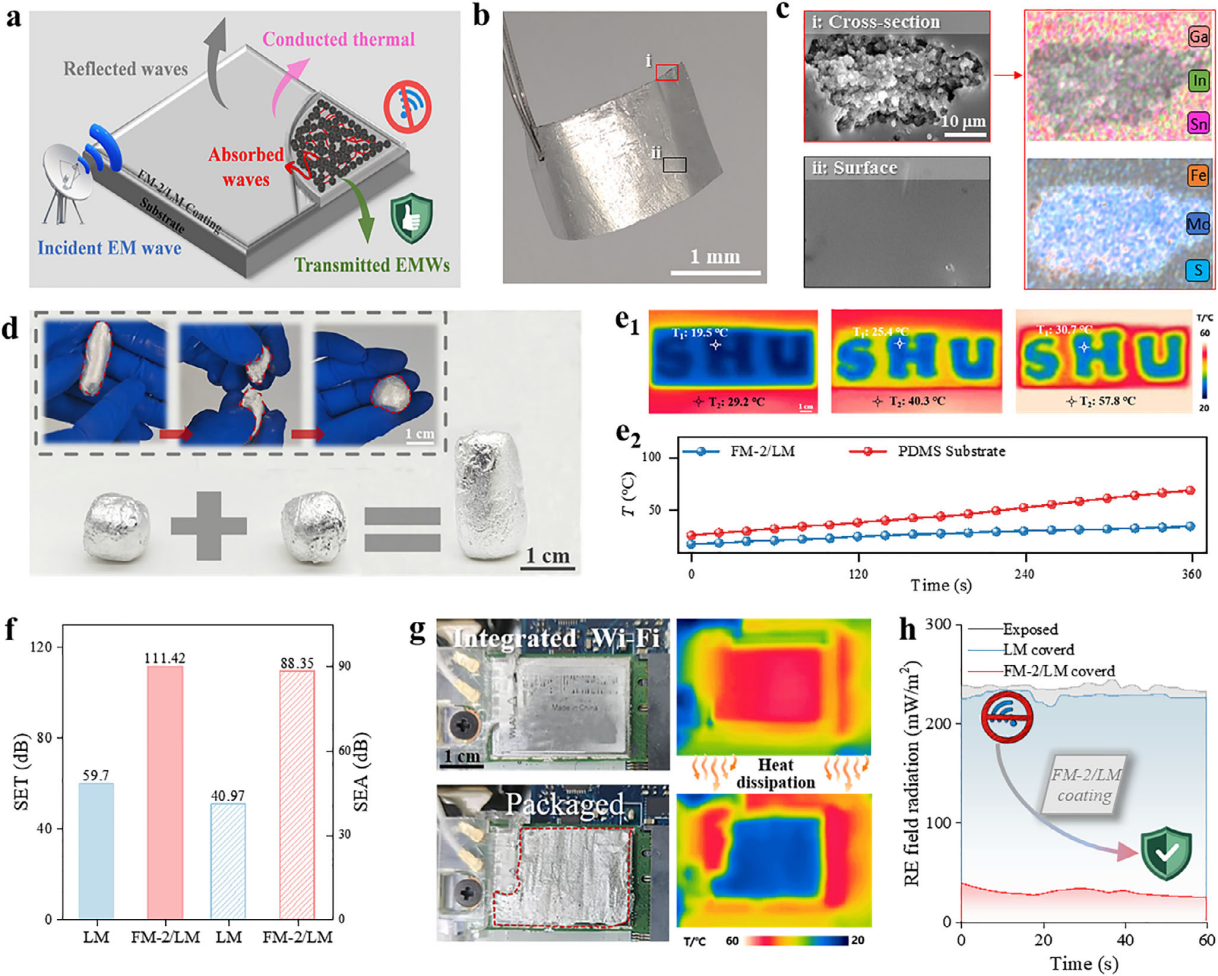
Extended application of magnetic‐dielectric heterostructure chains in EM protection. (a) Schematic for the application of FM‐2 heterostructure chains filled liquid metal (FM‐2/LM) composite film in microelectronic components. (b) Digital images of the whole FM‐2/LM composite film. (c) SEM and EDS mapping results for the cross‐section of the FM‐2/LM composite film. (d) Photographs of the FM‐2/LM composites for showing their operability. (e_1_) Infrared thermal images of the FM‐2/LM composites with designed shapes on the PDMS substrate, and (e_2_) recorded temperature variation during heating. (f) Comparison of EMI between pure LM and FM‐2/LM composite film. (g) Digital and infrared thermal images of integrated WLAN receivers before and after packaging with FM‐2/LM composite film. (h) The radiation values detected by mobile communication equipment under different covers.

To meet the growing demands for multifunctional EM materials in microelectronics, powder‐type EM absorbers are often used as fillers in suitable matrices to fabricate practicable composite. Distinct from traditional flexible polymer substrate, liquid metal (LM) possessed superior compatibility for accommodating absorber powders and excellent thermal conductivity for addressing the heat accumulation during EM attenuation process [61, 62, 63]. Thus, we engineered a multifunctional composite film with a thickness of 0.3 mm by incorporating the optimum FM‐2 absorbers into a EGaInSn‐based LM matrix via mechanical stirring and blade coating for investigating the practical EM protection application (Figure 6a, the fabrication procedure details were in the Experimental section of Supporting formation). Cross‐sectional SEM images identified that the original chain‑like morphology of the FM filler was preserved in the composite film (region i in Figure 6b,c), and EDS mapping verified the uniform distribution of all constituent elements (Figure S14).

Moreover, the incorporation of FM‐2 absorbers not only retained the LM's inherent self‐healing and malleability but also improved its adhesion and wettability on target surfaces, ultimately yielding a composite film with excellent processability for diverse EM functional applications (Figure 6d; Figure S15). Even under the mechanical distortion, the FM‐2/LM composite film could also solidly adhere on the target substrate (Figure S16). Although LM mainly behaved as viscous fluids without conventional solid‐like mechanical properties [63], the addition of FM‐2 absorbers effectively solidifies the LM to form a plasticine‐like state. Accordingly, the FM‐2/LM film could exhibit superior mechanical properties, as manifested by over 2‐fold tensile strength enhancement compared to that of the target substrate, and also overperformed the pure LM (Figure S16a). The FM‐2/LM film could readily withstand routine mechanical manipulations, including folding, rolling, twisting, and bending [22, 64]; and only minor cracks were observed on the film surface without any fracture after undergoing 1000 cycles bending treatment (Figure S16b,c). These mechanical property evaluations strongly confirmed the satisfying flexibility and toughness of the FM‑2/LM composite film.

Then, infrared thermal imaging results revealed a minimal surface temperature variation on the tested substrate covered with FM‐2/LM composite film under heating (Figure 6e), confirming its stable heat conductivity. Based on its superior plasticity and thermal management capability, the composite film was packaged onto an integrated WLAN receiver (Figure 6g). After packaging, the operating temperature increase of the receiver was markedly suppressed. In addition, the evaluations of EM interference protection properties indicated that the shielding effectiveness of the FM‐2 filled LM composite film was enhanced by 2 folds of that in the pure LM (Figure 6f). These impressive results demonstrated the application potential of the FM‐2/LM composite film in the integrated EM–thermal management of microelectronics. Beyond application in electronic components, this composite material was amenable to scalable fabrication for external EM protection in mobile electronic devices. The temporal evolution of radiation power during a call illustrated that a reduction efficiency of 86.7% against mobile phone radiation was achieved by the FM‐2/LM composite film (Figure 6h; Figure S17), further confirming its consistent and effective EM protection properties. These findings highlighted the promising potential of the FM‐2/LM composites in flexible wearable electronics and stealth technologies.

## 3 Conclusion

In summary, HSMF‐driven dual‐low‐dimensional engineering was developed to fabricate 1D magnetic‐dielectric Fe_3_O_4_@MoS_2_ heterostructure chains with tunable lengths, where the length could be precisely regulated from micrometer to millimeter scale by switching the applied HSMF. In addition, both their lengths and surface lattice defects exhibited a volcano‐shaped variation tendency along the increased strength and reached to the maximum at HSMF strength of 2 T. The increased uniaxial anisotropy endowed the 1D magnetic–dielectric Fe_3_O_4_@MoS_2_ heterostructure chains with a sensitive magnetic response, effectively enhancing the magnetic loss property. Meanwhile, owing to the tight encapsulation of the 2D MoS_2_ nanosheets on the ultralong 1D magnetic chain, abundant and successive magnetic–dielectric heterointerfaces were yielded for inducing interface polarization, further contributing to the dielectric loss mechanism. Based on the strengthened magnetic–dielectric synergy, the Fe_3_O_4_@MoS_2_ heterostructure chain with a recorded length of 2485.15 µm exhibited a broadband EM absorption performance with a EAB covered 5.5 GHz, and its filled liquid metal composite also presented over 86.7% radiation reduction efficiency and stable thermal conductivity. These findings provide significant guidance for designing high‐performance 1D magnetic–dielectric heterostructures and the application of EM functional materials in electronic devices.

## Conflicts of Interest

The authors declare no conflicts of interest.

## Supporting information




**Supporting File**: advs75440‐sup‐0001‐SuppMat.docx.

## Data Availability

The data that support the findings of this study are available from the corresponding author upon reasonable request.
