# Supplementary material for: Tailoring Successive Magnetic‐Dielectric Synergy for Enhancing Electromagnetic Absorption in Ultralong Heterostructure Chains Assisted by High Static Magnetic Field
Source: Adv Sci (Weinh). 2026 Apr 23;13(40):e75440. doi: 10.1002/advs.75440 (PMC13335691; doi:10.1002/advs.75440)
Supplement: Supplementary file 1 — Supporting File: advs75440‐sup‐0001‐SuppMat.docx. [file ADVS-13-e75440-s001.docx]

Supporting Information

**Tailoring Successive Magnetic-Dielectric Synergy for Enhancing Electromagnetic Absorption in Ultralong Heterostructure Chains Assisted by High Static Magnetic Field**

*Zhuolin Li, Meilong Feng, Wenbo Gao, Tianxiang Zheng*, Wenhao Lin* and Yunbo Zhong**

State Key Laboratory of Materials for Advanced Nuclear Energy & School of Materials Science and Engineering, Shanghai University, 200444, Shanghai, China.

*Corresponding authors’ E-mail: ztx@shu.edu.cn (T.X. Zheng), wenhaol@shu.edu.cn (W.H. Lin) and yunboz@staff.shu.edu.cn (Y.B. Zhong).

*Corresponding authors’ ORCID: 0000-0002-2081-3282 (T.X. Zheng), 0009-0003-1039-6408 (W.H. Lin), 0000-0002-0067-1299 (Y.B. Zhong).

**Experimental Section**

**Chemicals**

Anhydrous iron chloride (FeCl_3_, 97%), Sodium acetate trihydrate (CH_3_COONa, 99%), thiourea (CH_4_N_2_S, 99%), ethylene glycol (EG, 99%) and polyethylene glycol (PEG, 99%) were purchased from Sinopharm Chemical Reagent Co., Ltd. Ammonium molybdate tetrahydrate ((NH_4_)_6_Mo_7_O_24_⋅4H_2_O, 99%) were purchased from Beijing Vokai Biotechnology Co., Ltd. The EGaInSn (LM, containing 68.5% Ga, 21.5% In and 10.0% Sn, $\geq$99.99%) were purchased from China National Building Material Group Co., Ltd. Deionized (DI) water was used for all experiments in this study. All reagents and chemicals were not subjected to further purification.

**Synthesis of Fe_3_O_4_ microspheres**

Fe_3_O_4_ microspheres were prepared by a hydrothermal method. First, 5 mmol FeCl_3_ was dissolved in 40 ml EG solution, followed by the addition of 3.6 g CH_3_COONa and 1.0 g PEG under constant stirring 30 min to obtain a homogenous solution. Then, the mixture was transferred into a 50 ml Teflon-lined stainless-steel autoclave, sealed and maintained at 200 ℃ for 12 h. After the autoclave was naturally cooled to room temperature, the black products were collected and washed with absolute ethanol and DI water, and dried at 70 °C for 10 h.

**Synthesis of 1D Fe_3_O_4_@MoS_2_ heterostructure chain**

Fe_3_O_4_@MoS_2_ chains were fabricated through hydrothermal synthesis under the high static magnetic field (HSMF). Firstly, 0.132 g (NH_4_)_6_Mo_7_O_24_⋅4H_2_O and 0.286 g thiourea at ratio of about 1:2.17 were dissolved in 40 ml DI water and stirred. Until the mixture was completely dissolved, 0.1 g Fe_3_O_4_ microspheres were added into this solution and dispersed under ultrasonic treatment. After ultrasonic treatment for 15 min, the mixture was transferred into a 50 mL Teflon-lined stainless-steel autoclave and then put it in the HSMF equipment. Then, it was heated to 200 °C for 12 h under different magnetic field strength. When the temperature was naturally cooled to room temperature, the magnetic field was turned off and its strength gradually weakened until disappearance, after that, the black products were filtered and washed by DI water and absolute ethanol, and finally dried at 80 °C in an oven.

**Synthesis of FM-2/LM composite film**

First, a predetermined amount of the as-prepared ultralong magnetic-dielectric heterostructure chains (FM-2) was mixed with flowing EGaInSn-based liquid metal (LM) at a weight ratio of 1:9. The mixture was stirred using a mechanical agitator to obtain a shape-controlled, plasticine-like FM-2/LM composite. Then, the preformed composite was shaped into a uniform FM-2/LM thin film with controlled thickness using a blade coating ^[S1]^. The thickness of the final FM-2/LM composite film was measured as 0.30 mm using a vernier caliper (precision: ± 0.02 mm).

**Material Characterization**

The phase composition of samples was analyzed using X-ray diffraction (XRD) on a multifunctional X-ray diffractometer equipped with a Cu Kα radiation source (18 kW, 20.0 - 40.0 kV, 10.0 - 450.0 mA). The surface morphologies and microstructures were observed using a scanning electron microscopy (SEM, VEGA 3 Easy Probe) equipped with energy dispersive spectrometer. The refined structure and crystallographic information were acquired using a field-emission transmission electron microscope (JEOL JEM-2100F). X-ray photoelectron spectroscopy (XPS) were performed on a Thermo Scientific ESCALAB 250Xi spectrometer with a monochromatic X-ray source. The hysteresis loops were analyzed using a vibrating sample magnetometer (LakeShore 7407).

**Electromagnetic wave absorption measurement**

According to the coaxial line method, a vector network analyzer (Agilent, E5071C) was used to measure the electromagnetic (EM) wave absorption performance and electromagnetic parameters of the Fe_3_O_4_@MoS_2_. EM parameters (complex permittivity and permeability) were obtained in the frequency range of 2–18 GHz. The composite materials (40%) were evenly mixed with molten paraffin and molded into a coaxial ring of 7.00 mm outer diameter and 3.04 mm inner diameter. According to the transmission line theory, the reflection loss (RL) values of samples calculated from the measured electromagnetic parameters^[S2]^. The equations are as follows:

$Z_{\text{in }}=Z_{0}\sqrt{\frac{\mu_{r}}{\varepsilon_{r}}}\tanh\left[ j\left( \frac{2\pi fd}{c} \right)\sqrt{\mu_{r}\varepsilon_{r}} \right]$ (S1)

$RL=20\log\left| \frac{Z_{in}-Z_{0}}{Z_{in}+Z_{0}} \right|$ (S2)

Where $Z_{in}$ and $Z_{0}$ are the input resistance and impedance of free space, respectively; $\mu_{r}$ and $\varepsilon_{r}$ are the relative complex permittivity and permeability, respectively; $f$ is the frequency of incident EM wave; $d$ is the thickness of specimen; $c$ is the velocity of electromagnetic waves in a vacuum.

The attenuation constant (α) can be calculated through the equation S3:

$\alpha=\frac{\sqrt{2}\pi f}{c}\sqrt{\left( \mu^{''}\varepsilon^{''}-\mu^{'}\varepsilon^{'} \right)+\sqrt{{{(\mu}^{'}\varepsilon^{''}+\mu^{''}\varepsilon^{'})}^{2}+{{(\mu}^{''}\varepsilon^{''}-\mu^{'}\varepsilon^{'})}^{2}}}$ (S3)

The eddy current loss factor ($C_{0}$) can be expressed via equation S4:

$C_{0}=\mu^{''}\left( \mu^{'} \right)^{-2}f^{-1}$ (S4)

**Electromagnetic interference (EMI) shielding performance measurements:**

The EMI parameters of FM-2/LM were measured with a vector network analyzer (E5071C, Agilent, USA) within 8.2 to 12.4 GHz, this frequency range is usually rectangular waveguide sample holder. The EMI SE represents the ability of samples to attenuate the energy of the incident EM waves. EMI SE is defined as the logarithm of the ratio of the incident power to the transmitted power. These following equations are used to calculate total EMI SE (${SE}_{T}$), reflection efficiency (${SE}_{R}$), absorption efficiency (${SE}_{A}$), and multiple reflection efficiency (${SE}_{M}$).

${SE}_{T}=10log\frac{1}{T}$ (S5)

${SE}_{R}=10log\left( \frac{1}{1-R} \right)$ (S6)

${SE}_{A}=10log\left( \frac{1-R}{T} \right)$ (S7)

${SE}_{T}= {SE}_{R}+ {SE}_{A}+ {SE}_{M}$ (S8)

When ${SE}_{T} >10 dB$ the ${SE}_{M}$ could be ignored ^[S1]^. To further compare the effectiveness of the shielding materials, the EMI SE is normalized to vanish the effects of thickness and calculated as EMI SE/*t*, where *t* is the thickness of the material.

**Micromagnetic simulation**

All micromagnetic simulations were conducted with the open-source Mumax3 software, leveraging its intrinsic GPU acceleration ^[S3]^. Guided by the Landau-Lifshitz-Gilbert equation and the principle of minimum energy, the simulations were implemented via a finite-difference algorithm to model dynamic processes and solve 3D structures. In this research, the simulation is used to analyze the varieties of magnetic energy in four models FM-x and the dynamic spin structure. The model of FM-0 was constituted with randomly scattered microspheres of diameter 200 nm, and the model of FM-x (x = 1, 2, 3) individual microchains were composed with aligned microspheres of diameter 200 nm, FM-1 medium-chain were made with eight microspheres of diameter 200 nm, FM-2 long-chain comprised with eleven microspheres of diameter 200 nm, FM-3 short-chain formed from five microspheres of diameter 200 nm. The following magnetic parameters were defined in the simulation for visualization: the value of saturation magnetization is 4.17×10^5^ A⋅m^-1^, micromagnetic exchange constant 1.2×10^-11^ J⋅m^-1^, and magneto-crystalline anisotropy constant 2.0×10^5^ J⋅m^-3^. The magnetic field densities change according to the variations of the FM-x samples in the simulation.

**Radar cross-section (RCS) simulation**

Generally, for a specified scattering source, the RCS value ($\sigma$) was calculated by theta and phi in spherical coordinates as follow function:^[S2]^

$\sigma\left( dBm^{2} \right)=10log \left[ \frac{4\pi S}{\lambda^{2}} \left| \frac{E_{S}}{E_{i}} \right|^{2} \right]$ (S9)

Where *S*, $\lambda$, $E_{S}$, and $E_{i}$ are area of the simulated plate, the length of the incident EM wave, the electric field intensity of transmitting waves, and the incident field intensity of accepting wave, respectively.

To access the practical application potential of FM-x dual low-dimension heterostructure, CST STUDIO SUITE 2018 software is employed to simulate the RCS of a rectangular ultrathin perfect electric conductor (PEC) plate substrate (size: 200×200 mm) coated with the same thickness of FM-x specimens, respectively. This model defines the EM incidence direction as the positive z-axis (θ = 90º). The color and structure of the radiation lobes map the scattering intensity, which serves as a direct measure of absorption performance is lower scattering implies stronger absorption. To evaluate RCS reduction performance, the three-dimensional scattering patterns of a pristine PEC plate and FM-x coated plates were simulated at the frequency of 10.6 GHz, across angular sweeps from 0° to 180°.

**Figures and Tables**


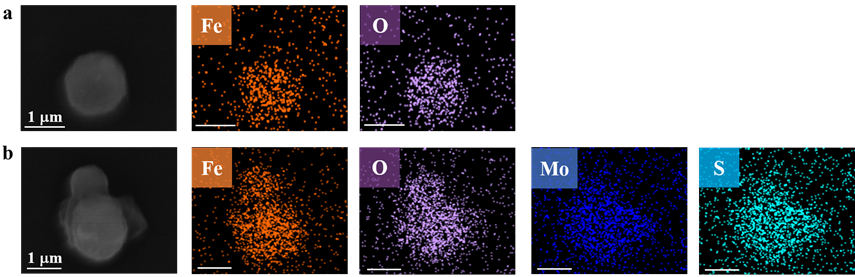


**Figure S1** SEM images and EDS mapping of a) Fe_3_O_4_ magnetic unit and b) Fe_3_O_4_@MoS_2_.


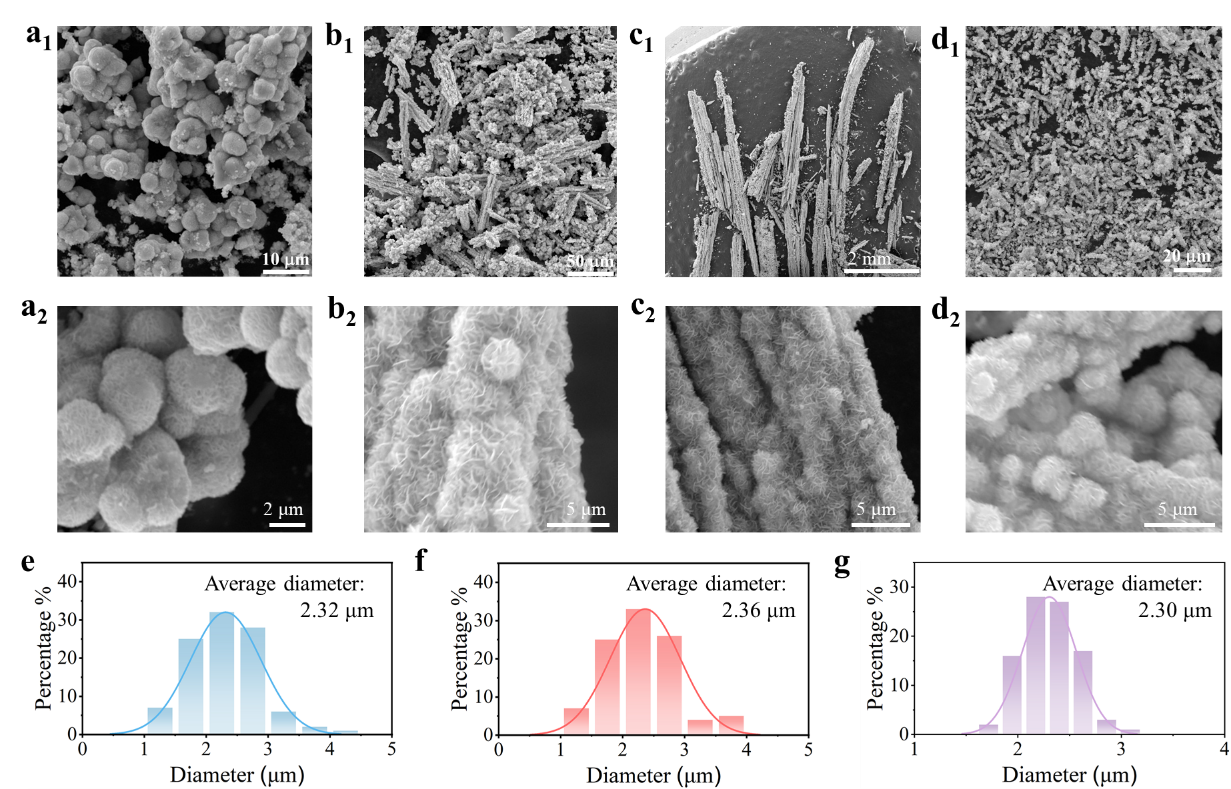


**Figure S2** SEM images of overall and local FM-x specimens. a_1-2_) FM-0, b_1-2_) FM-1, c_1-2_) FM-2, d_1-2_) FM-3. The diameters distribution statistics of e) FM-1, f) FM-2, and g) FM-3. The FM-0 sample is the sphere-like structure, its diameter equals to the size, as shown in the inset of Figure b_1_.


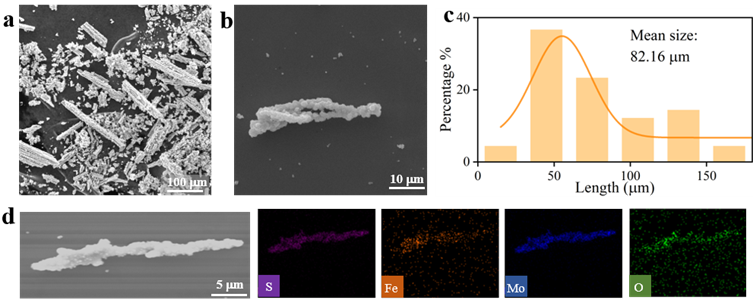


**Figure S3** a-b) SEM images of Fe_3_O_4_@MoS_2_ chain synthesized under 4 T. c) Corresponding chain length statistic and d) elements mapping.


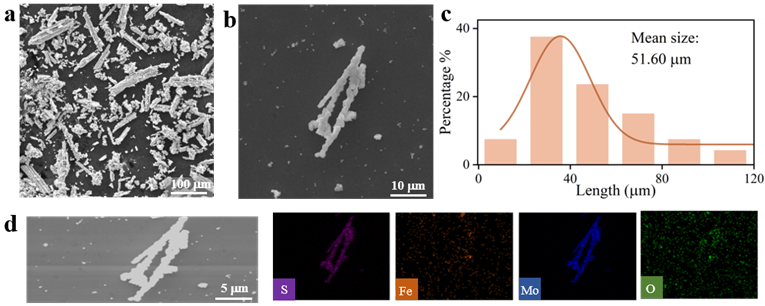


**Figure S4** a-b) SEM images of Fe_3_O_4_@MoS_2_ chain synthesized under 6 T. c) Corresponding chain length statistic and d) elements mapping.

**
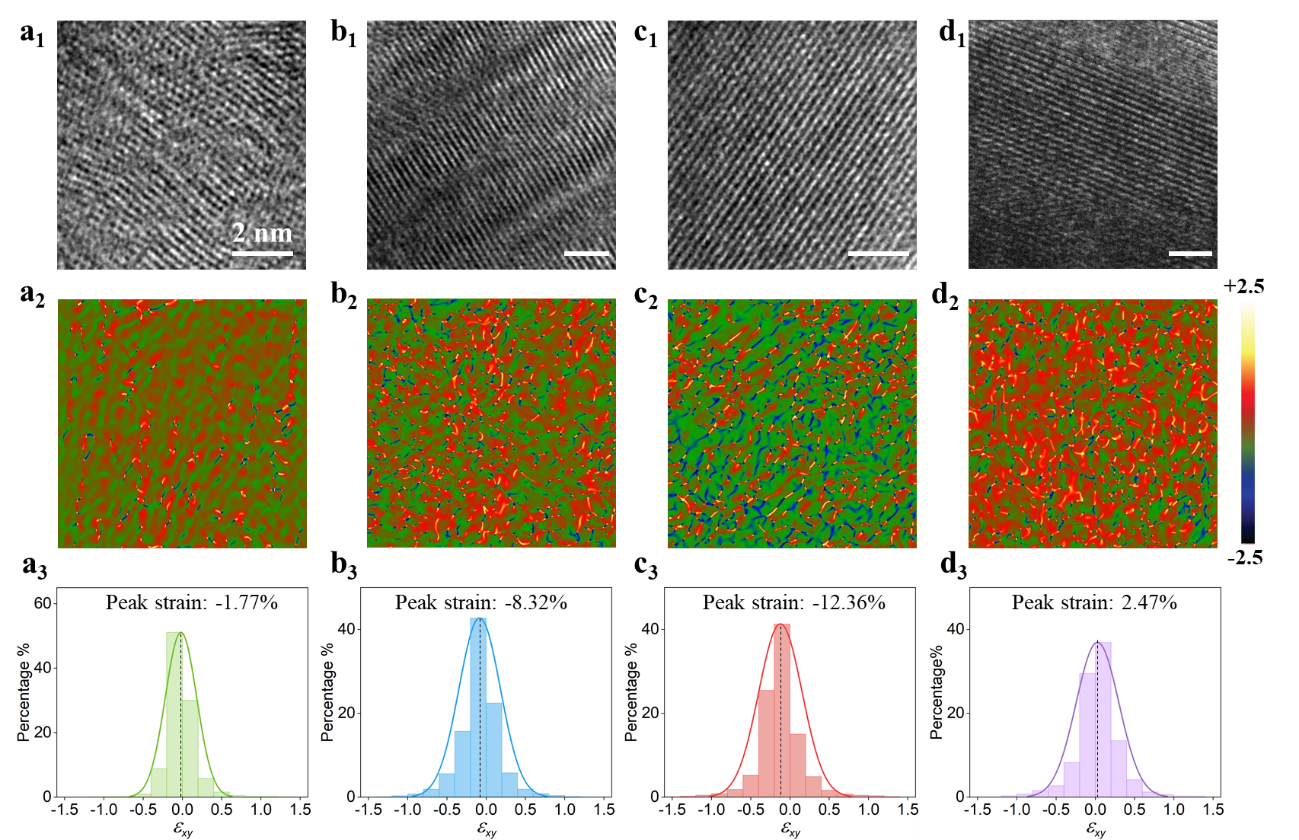
**

**Figure S5** HRTEM images, strain field micrographs and corresponding histogram of strain distribution of specimen a_1-3_) FM-0, b_1-3_) FM-1, c_1-3_) FM-2, d_1-3_) FM-3.


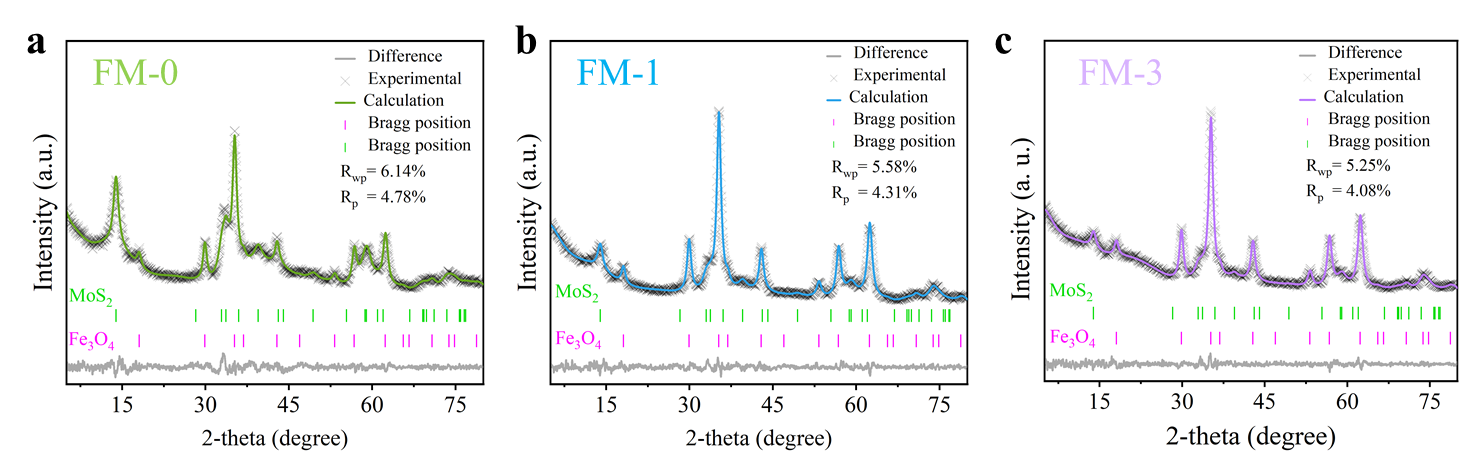


**Figure S6** Refined XRD patterns of FM-x samples. a) FM-0, b) FM-1, c) FM-3.


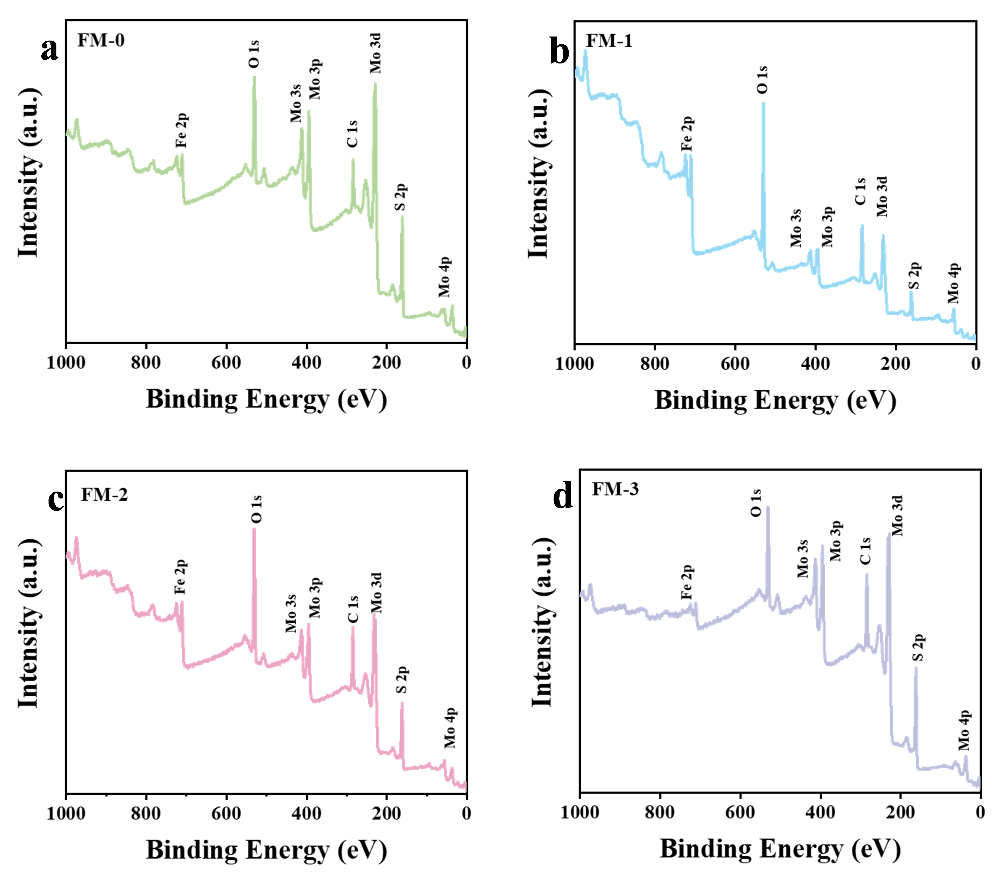


**Figure S7** XPS spectra surveys of FM-X specimens. a) FM-0, b) FM-1, c) FM-2, d) FM-3.


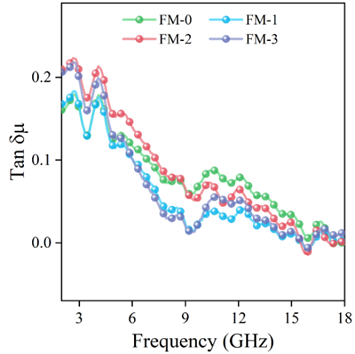


**Figure S8** Tangent value of magnetic loss of FM-x samples.

**
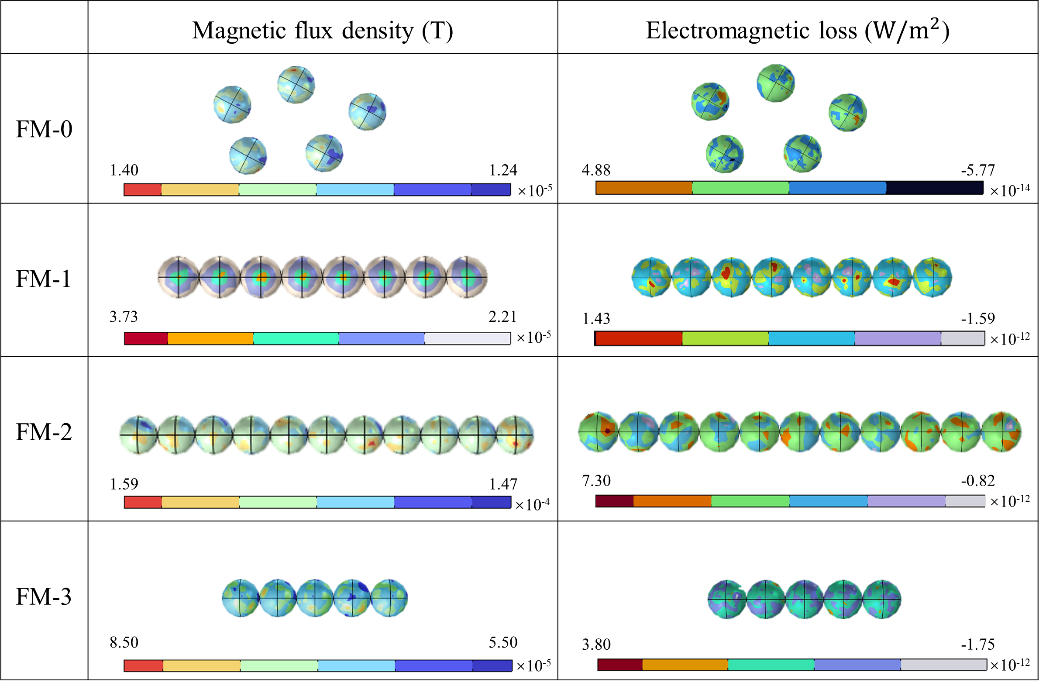
**

**Figure S9** Finite elements simulated magnetic flux density and EM loss values of FM-x specimens.


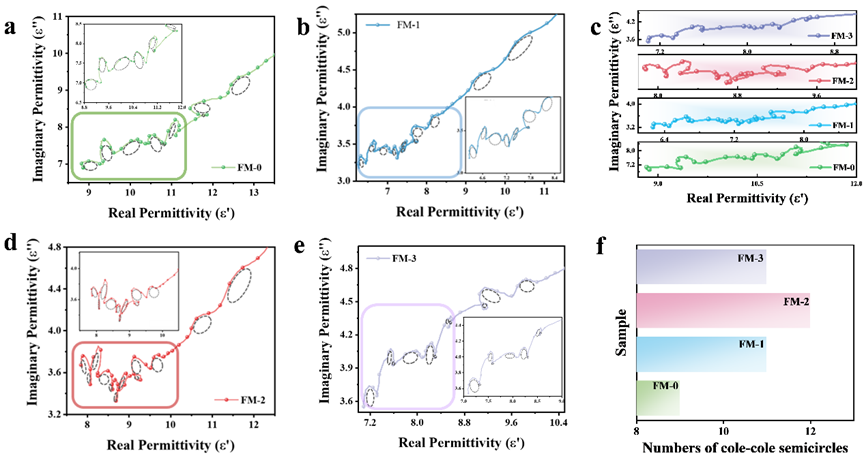


**Figure S10** Cole-Cole curves of a) FM-0, b) FM-1, d) FM-2, e) FM-3. c) Enlarged Cole-Cole curve. f) Numbers of Cole-Cole semicircles in FM-x samples of FM-x specimens.


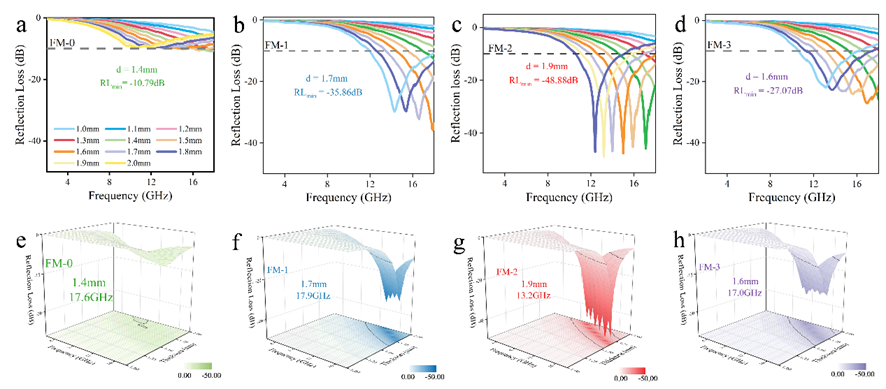


**Figure S11** 1D and 3D RL results of a, e) FM-0, b, f) FM-1, c, g) FM-2, d, h) FM-3.


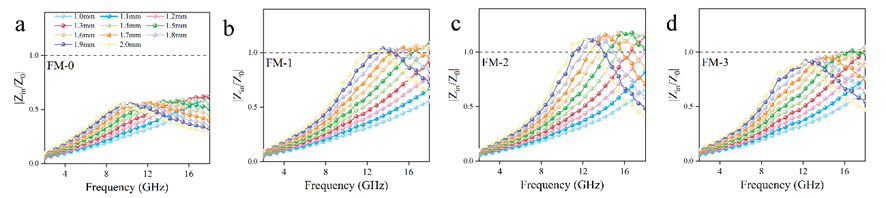


**Figure S12** $\left| Z_{in}/Z_{0} \right|$ curves of a) FM-0, b) FM-1, c) FM-2, d) FM-3 at different thickness.


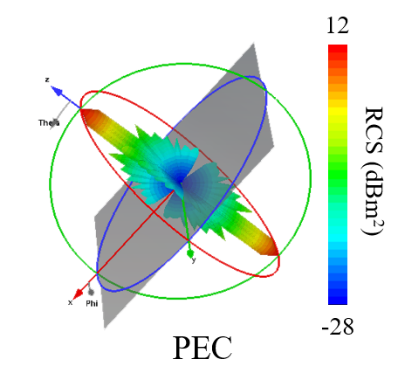


**Figure S13** 3D RCS patterns of pure PEC plate.


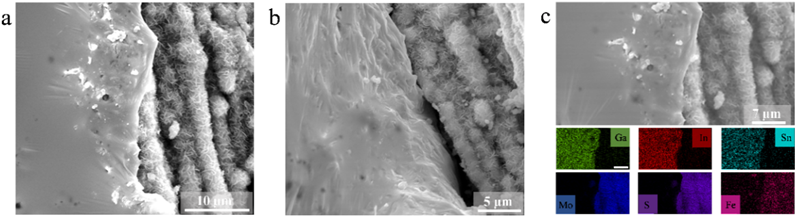


**Figure S14** a, b) SEM images and c) EDS mapping results of the FM-2/LM composite film.


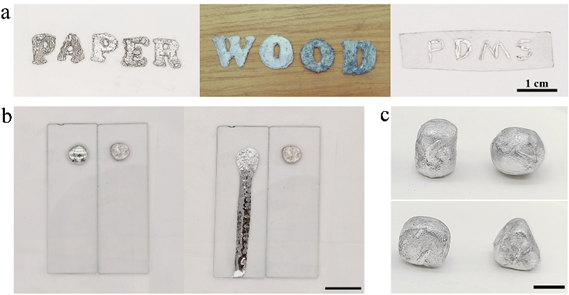


**Figure S15** a) Wettability of the FM-2/LM composites for different materials. Apply FM-2/LM to the paper, wood and PDMS. b) Digital photos showed the adhesion of pure LM (the left) and FM-2/LM composites (the right) on flat glass and tilted glass plates. c) Photographs of the FM-2/LM composites with different shapes, showing its malleability.


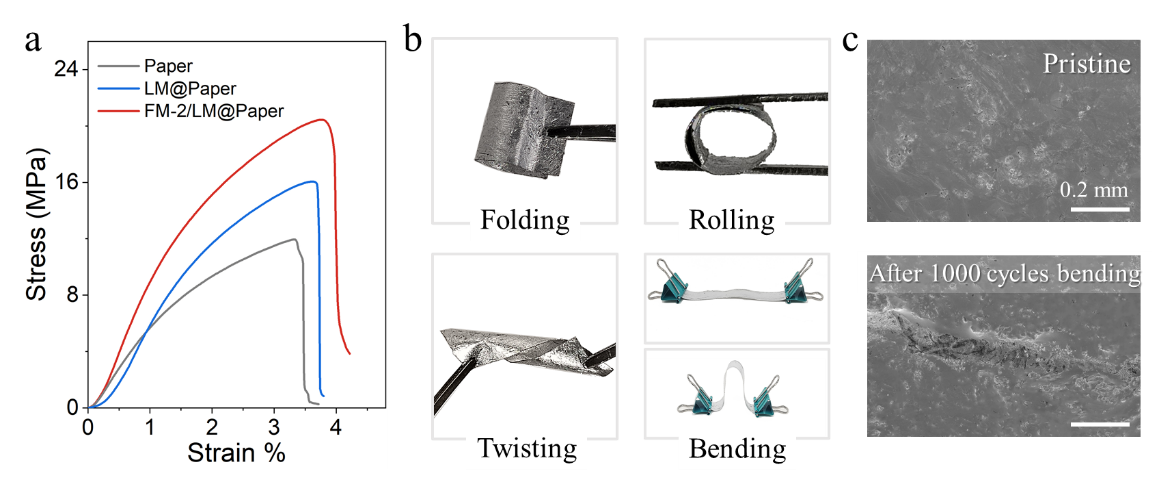


**Figure S16** Mechanical properties analysis. a) Stress-strain curves of the commercial paper, and it coated with pure LM and FM-2/LM composite film, and the coating thicknesses were 0.3 mm. b) Digital photographs of FM-2/LM composite film undergoing folding, rolling, twisting, and bending. c) SEM images of FM-2/LM composite film before and after undergoing 1000 bending cycles, the scale bars are 0.2 mm.

**
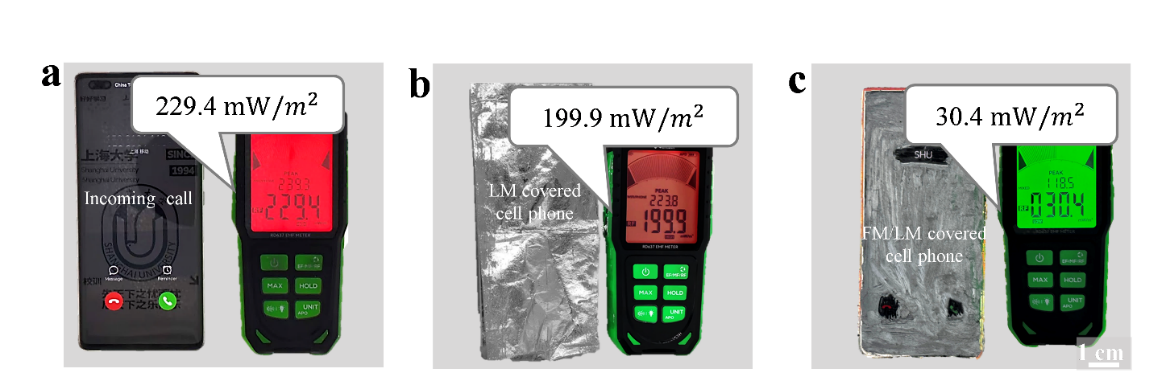
**

**Figure S17** EMI shielding tests of mobile phones covered by a) nothing, b) paper coated with pure LM, c) FM-2/LM composite with the same thickness in pure LM.

**Table S1** Chain length comparison of reported 1D structure and this work.

| Samples | Average length (μm) | diameter (μm) | Aspect ration | Ref. |
| --- | --- | --- | --- | --- |
| CoNi | 24 | 2 | 12 | S4 |
| Co/Cu | 200 | 10 | 20 | S5 |
| CoNi | 30 | 2 | 15 | S6 |
| Co@Fe_x_Co_1-x_ | 220 | 0.4 | 550 | S7 |
| Co | 25 | 1 | 25 | S8 |
| Fe_3_O_4_@SiO_2_ | 10 | 0.2 | 50 | S9 |
| Fe_3_O_4_@void@SiO_2_@PPy | 30 | 0.5 | 60 | S10 |
| Fe_3_O_4_@TiO_2_ | 10 | 0.4 | 25 | S11 |
| ZnFe_2_O_4_@SiO_2_@C@NiCo_2_O_4_ | 20 | 0.16 | 125 | S12 |
| Fe_3_O_4_@void@mSiO_2_@MnO_2_ | 6 | 0.24 | 25 | S13 |
| Ni@PVP | 10 | 0.245 | 40.82 | S14 |
| Fe/Fe_3_C/C | 10 | 0.2 | 50 | S15 |
| Ni@PANI | 2 | 0.02 | 100 | S16 |
| Co@PANI | 12 | 0.33 | 36.36 | S17 |
| FeCo/PVP | 3 | 0.025 | 120 | S18 |
| FM-1 | 37.80 | 2.33 | 16.22 | This work |
| FM-2 | 2485.15 | 2.33 | 1066.59 | This work |
| FM-3 | 9.38 | 2.33 | 4.03 | This work |
| FM-4T | 82.16 | 2.33 | 35.26 | This work |
| FM-6T | 51.60 | 2.33 | 22.15 | This work |

**Table S2** Cell parameters of Fe_3_O_4_ and MoS_2_ in the FM-X from Rietveld refinement.

| Sample | Composition  (wt. %) | Bond length (Å) | | | | Bond angle (º) | | | Volume (Å^3^) |
| --- | --- | --- | --- | --- | --- | --- | --- | --- | --- |
|  |  | a | b | c | α | | β | γ |  |
| FM-0 | Fe_3_O_4_ (54.8%) | 8.3859 | 8.3859 | 8.3859 | 90 | | 90 | 90 | 589.728 |
|  | MoS_2_ (45.2%) | 3.1184 | 3.1184 | 12.5188 | 90 | | 90 | 120 | 105.426 |
| FM-1 | Fe_3_O_4_ (81.3%) | 8.3879 | 8.3879 | 8.3879 | 90 | | 90 | 90 | 590.165 |
|  | MoS_2_ (18.7%) | 3.1147 | 3.1147 | 12.5245 | 90 | | 90 | 120 | 105.228 |
| FM-2 | Fe_3_O_4_ (85.7%) | 8.3886 | 8.3886 | 8.3886 | 90 | | 90 | 90 | 590.297 |
|  | MoS_2_ (14.3%) | 3.0945 | 3.0945 | 12.5668 | 90 | | 90 | 120 | 104.215 |
| FM-3 | Fe_3_O_4_ (87.1%) | 8.3907 | 8.3907 | 8.3907 | 90 | | 90 | 90 | 590.736 |
|  | MoS_2_ (12.9%) | 3.1179 | 3.1179 | 12.5128 | 90 | | 90 | 120 | 105.346 |

**Table S3** EM absorption performance comparison between this work and reported analogous absorbers.

| Absorber | EAB (GHz) | RL_min_  (dB) | Thickness  (mm) | Ref. |
| --- | --- | --- | --- | --- |
| ZnFe_2_O_4_@PPy | 3.5 | -41.0 | 4.1 | S19 |
| ZnFe_2_O_4_@MoS_2_ | 1.88 | -31.29 | 5.2 | S20 |
| BaFe_11.6_Co_0.4_O_19_@Fe_3_O_4_ | 3.5 | -48.9 | 5.4 | S21 |
| C/Fe_3_O_4_ | 2 | -40.1 | 5.12 | S22 |
| MoS_2_@Fe_3_O_4_@PANI | 4 | -40.97 | 2.3 | S23 |
| Fe_3_O_4_/Fe@C@MoS_2_ | 2.24 | -53.79 | 4.4 | S24 |
| Fe/ Fe_3_O_4_@C@MoS_2_ | 4 | -36.1 | 5.4 | S25 |
| MoS_2_/Fe_3_O_4_ | 2 | -18.0 | 3.0 | S26 |
| CoNi/PDMS | 4.1 | -56.7 | 1.04 | S27 |
| Cu@Co | 3.2 | -43.5 | 7.3 | S28 |
| CoNi | 5.4 | -42.11 | 3.5 | S6 |
| Fe_3_O_4_@TiO_2_ | 5.09 | -21.29 | 3.0 | S11 |
| FM-2 | 5.5 | -48.88 | 1.8 | This work |

**References in Supporting Information**

1. H. Jiang, B. Yuan, H. Guo, F. Pan, F. Meng, Y. Wu, X. Wang, L. Ruan, S. Zheng, Y. Yang, Z. Xiu, L. Li, C. Wu, Y. Gong, M. Yang, W. Lu, *Nat. Commun.* **2024**, 15, 6138.
2. L. Zhou, P. Hu, M. Bai, N. Leng, B. Cai, H. Peng, P. Zhao, Y. Guo, M. He, G.S. Wang, J. Gu, *Adv. Mater.* **2025**, 37, 2418321.
3. J. Leliaert, J. Mulker, *J. Appl. Phys.* **2019**, 35, 663.
4. M. He, J. Hu, H. Yan, X. Zhong, Y. Zhang, P. Liu, J. Kong, J. Gu, *Adv. Funct. Mater.* **2025**, 35, 2316691.
5. Y. Lu, W. Shao, L. Wu, L. Liu, G. Tong, W. Wu, *J. Alloys Compd.* **2020**, 847, 156509.
6. M. Qiao, J. Li, S. Li, D. Wei, X. Lei, W. Lei, J. Wei, Q. Zhang, M. Ma, *J. Alloys Compd.* **2022**, 926, 166854.
7. Y. Han, H. Guo, H. Qiu, J. Hu, M. He, X. Shi, Y. Zhang, J. Kong, J. Gu, *Adv. Funct. Mater.* **2025**, 35, 2506803.
8. W. You, K. Pei, L. Yang, X. Li, X. Shi, X. Yu, H. Guo, R. Che, *Nano Res.* **2020**, 13, 72.
9. T. Zhang, Q. Yue, P. Pan, Y. Ren, X. Yang, X. Cheng, F. Alharthi, A. Alghamdi, Y. Deng, *Nano Res.* **2021**, 14, 4197.
10. M. Qiao, D. Wei, X. He, X. Lei, J. Wei, Q. Zhang, *J. Mater. Sci.* **2021**, 56, 1312.
11. M. Qiao, J. Wang, D. Wei, J. Li, X. Lei, W. Lei, J. Wei, Q. Zhang, *Mater. Today Nano*. **2022**, 18, 100203.
12. M. Ma, W. Li, Z. Tong, Y. Ma, Y. Bi, Z. Liao, J. Zhou, G. W, M. Li, J. Yue, X. Song, X. Zhang, *J. Colloid Interf. Sci.* **2020**, 578, 58.
13. M. Qiao, J. Li, D. Wei, X. He, X. Lei, J. Wei, Q. Zhang, *Micropor Mesopor Mater*. **2021**, 314, 110867.
14. J. Liu, M. Cao, Q. Luo, H. Shi, W. Wang, J. Yuan, *ACS Appl. Mater. Interfaces.* **2016**, 8, 22615.
15. J. Sun, Z. He, W. Dong, W. Wu, G. Tong, *J. Alloys Compd.* **2019**, 782, 193-202.
16. Y. Li, J. Wang, H. Li, B. Zhang, Y. Cui, J. Cai, Y. Wang, Y. Zhang, Z. Bao, Y. Zhang, Y. Wu, *J. Alloys Compd.* **2020**, 821, 153531.
17. B. Dai, Y. Ma, S. Feng, H. Wang, M. Ma, J. Ding, X. Yin, T. Li, *J. Colloid Interface Sci.* **2022**, 627, 113.
18. X. Zhang, Y. Li, R. Liu, Y. Rao, H. Rong, G. Qin, *ACS Appl. Mater. Interfaces.* **2016**, 8, 3494.
19. Z. Li, H. Zhu, L. Rao, M. Huang, Y. Qian, L. Wang, Y. Liu, J. Zhang, Y. Lai, R. Che, *Small*. **2024**, 20, 2308581.
20. J. Xiao, X. Qi, X. Gong, Q. Peng, Y. Chen, R. Xie, W. Zhong, *J. Mater. Sci. Technol.* **2023,** 139, 137.
21. M. Yuan, B. Zhao, C. Yang, K. Pei, L. Wang, R. Zhang, W. You, X. Liu, X. Zhang, R. Che, *Adv. Funct. Mater.* **2022**, 32, 2203161.
22. C. Xu, K. Luo, Y. Du, X. Lv, C. Zhang, W. You, H. Cheng, R. Che, *Adv. Funct. Mater.* **2025**, e12806.
23. W. Zhang, L. Li, W. Zhu, H. Yan, S. Qi, *Mater. Sci.: Mater. Electron.*, **2017**, 28(20): 15488-15494.
24. Z. Tong, Z. Liao, Y. Liu, M. Ma, Y. Bi, W. Huang, Y. Ma, M. Qiao, G. Wu, *Carbon.* **2021**, 179, 646.
25. Y. Liu, C. Tian, F. Wang, B. Hu, P. Xu, X. Han, Y. Du, *Chem. Eng. J.* **2023**, 461, 141867.
26. L. Jin, Y. Zheng, X. Liu, Y. Zhang, Z. Li, Y. Liang, S. Zhu, H. Jiang, Z. Cui, and S. Wu, *Small*. **2022**, 18, 2204028.
27. M. He, J. Hu, H. Yan, X. Zhong, Y. Zhang, P. Liu, J. Kong, J. Gu, *Adv. Funct. Mater.* **2025**, 35, 2316691.
28. B. Zhao, Y. Li, Q. Zeng, L. Wang, J. Ding, R. Zhang, R. Che, *Small.* **2020**, 16, 2003502.
